# Topical Treatment Is Effective and Safe for Acute Ankle Sprains: The Multi-Center Double-Blind Randomized Placebo-Controlled TRAUMED Trial

**DOI:** 10.3390/jcm13030841

**Published:** 2024-02-01

**Authors:** Ludger Gerdesmeyer, Johannes Vester, Christian Schneider, Britt Wildemann, Christine Frank, Myron Schultz, Bernd Seilheimer, Alta Smit, Gino Kerkhoffs

**Affiliations:** 1 Orthopedics and Trauma Surgery, Kiel Municipal Hospital, 24116 Kiel, Germany; 2 idv Data Analysis & Study Planning, 82131 Gauting, Germany; 3 Orthopedic Center Theresie, 80339 Munich, Germany; 4 Experimental Trauma Surgery, Jena University Hospital, Friedrich Schiller University Jena, 07747 Jena, Germany; 5 Heel GmbH, 76532 Baden-Baden, Germany; 6 Department of Orthopedic Surgery and Sports Medicine, Amsterdam Movement Sciences, Amsterdam University Medical Centers, University of Amsterdam, 1105 AZ Amsterdam, The Netherlands

**Keywords:** ankle sprain, injury, musculoskeletal pain, treatment, anti-inflammatory agents, TRAUMED, traumeel, Tr14, diclofenac

## Abstract

Background: Topical NSAIDs are widely used to treat ankle sprains. Traumed (Tr14) gel is a multicomponent formulation, demonstrating inflammation-resolution properties. Methods: This multicenter, double-blind trial investigated the efficacy and safety of Tr14 gel versus placebo gel and non-inferiority versus 1% diclofenac gel, applied 3×/day for 7 days after acute lateral ankle sprain (EudraCT Number: 2016-004792-50). The primary outcome was AUC for pain on passive movement, assessed by VAS from baseline to Days 4 and 7. Results: The trial population included 625 patients aged 18 to 78 years. The AUC scores were 187.88 and 200.75 on Day 4 (*p* = 0.02) and 294.14 and 353.42 on Day 7 (*p* < 0.001) for Tr14 and placebo, respectively. For Tr14 compared to diclofenac, the AUC scores were 187.50 and 197.19 on Day 4 (*p* = 0.3804) and 293.85 and 327.93 on Day 7 (*p* = 0.0017), respectively. On the FAAM-ADL subscale, Tr14 was superior to placebo and non-inferior to diclofenac at all time points. Time to 50% pain improvement was lowest for Tr14 (6.0 days), compared to placebo (7.1 days) and diclofenac (7.0 days). Adverse events were uncommon and minor. Conclusions: Tr14 gel is effective and safe in acute ankle sprains, compared to placebo gel and diclofenac gel, and has faster pain resolution. Trial registration: The trial was registered in clinicaltrialsregister.eu, EudraCT number 2016-004792-50 on 07.06.2017.

## 1. Introduction

Acute ankle sprains are common, disabling, and of high clinical relevance, accounting for more than 1 million medical attendances per year worldwide [1]. They are associated with an appreciable socio-economic burden [2]. Approximately 16–40% are sports-related, where time to recovery is important for athletes, professionals, and those training for major competitions. Lateral ankle sprains are the most prevalent (85%), particularly affecting the anterior talofibular ligament. This is usually secondary to a high-velocity inversion and internal rotation injury [3]. Some 40% of ankle sprains are at risk of chronicity. Typical features include pain on weight loading, ankle instability, and mobility issues [4,5,6].

Commonly employed therapies for ankle sprains (Grade 1: mild; Grade 2: moderate sprain/partial ligament lesion) include oral or topical analgesia, rest, ice, compression, elevation (RICE), functional support, and initiation of an exercise program. A minority with a Grade 3 injury (severe sprain/full ligament tear), notably athletes, may undergo surgical repair in the hope of optimizing healing. Guidelines are available. However, there is a general lack of good-quality trial data in support of many current treatments [4,6,7].

Oral NSAIDs are routinely recommended following musculoskeletal injuries because of their analgesic and anti-inflammatory effects [8]. Inhibiting PGI2, PGE2, and TXA2 appears to be central to their actions [9]. The use of oral NSAIDs is associated with a diverse side effect profile, affecting multiple organ systems, including gastrointestinal, cardiovascular, and renal complications [10]. Approximately 2% of users experience hypersensitivity reactions [11]. There is also evidence to suggest that this class of drugs increases the risk of acute pain transitioning to chronic pain and may delay or interfere with normal tissue healing, encourage harmful pain-masking, and predispose to re-injury and chronicity [12]. By comparison, the use of topical NSAIDs appears to be generally safe and effective and is now widely employed following acute ankle sprains, with diclofenac gel generally considered the ‘gold standard’ [13,14]. However, a downside associated with all formulations is the ecological risks of diclofenac entering the water cycle [15].

Many treatment options are widely used in the treatment of acute ankle sprains. Most options address mechanical stabilization and protection. Nowadays, the focus is much more on managing soft tissue inflammation in acute sprains. The paradigm of treating pain is changing slowly in favor of supporting inflammation resolution pathways in inflamed soft tissue rather than inhibiting the inflammation-associated pathways with nonsteroidal anti-inflammatory substances. In their Nature article, Serhan et al. (2014) reviewed the evidence suggesting that inflammation resolution is an active—not passive, as was thought before—process involving eicosanoid (prostaglandins and leukotrienes) metabolism [16]. This process is driven by the lipid mediators known as Specialized Proresolving Mediators or SPMs (lipoxins, resolvins, maresins, and others). They suggested that stimulating inflammation resolution with SPM-agonists could provide a more physiological treatment option. This approach is supported by translational research showing that NSAIDs can diminish SPM response while reducing COX-associated inflammatory signaling [17]. These findings suggest that acute proinflammatory signals are mechanistically linked to the induction of a biological active inflammatory resolution program. This is corroborated by recent research showing that neutrophil activation protects against the development of chronic pain [12]. Their depletion delays the resolution of pain, and NSAIDs may promote acute pain becoming chronic due to the potential inhibition of inflammation resolution. Therefore, the inclusion of inflammation-resolving drugs as a new treatment modality for inflammatory pain is currently being researched, and clinical trials are underway [18]. Tr14 gel is an established natural medicinal product consisting of 14 components (Supplementary Table S1). It is available without prescription in more than 50 countries to treat inflammation and pain caused by musculoskeletal injuries [19]. In preclinical studies, Tr14 demonstrated proresolving properties due to a modulating effect on proresolution signaling pathways and the behavior of multiple immune cell types involved in tissue repair [20,21]. This results in the increasing biosynthesis of specialized proresolving mediators and the shortening of the inflammation resolution index and the duration of inflammation [22]. Our research also suggests that Tr14 plays a role in modulating the neutrophil-macrophage axis, which seems to be the central part of the mechanistic difference between Tr14 and diclofenac [21].

Clinical trials and observational studies have confirmed the safety, efficacy, and effectiveness of Tr14 for the treatment of ankle sprains, tendinopathy, epicondylitis, and knee osteoarthritis [19]. Its value in the acute setting is potentially important for those seeking optimum fitness for whom a loss of 1–2 days of training could be detrimental [23]. The time of return to regular sports activities has a very high impact on professional sports and the Olympics. A 1-day advantage, for example, could affect the whole season for a major football club. Consequently, data supporting the use of Tr14 has resulted in a recommendation that it should be included in future treatment protocols following musculoskeletal injury [24].

Among the sports community, both topical diclofenac (inflammation-inhibiting) and Tr14 (inflammation-resolving) are widely used as topical treatment options. The evidence for diclofenac’s efficacy in treating ankle sprains and soft tissue injuries goes back to the 1980s [25,26,27]. Recent systematic reviews confirmed the efficacy of topical diclofenac applications for treating musculoskeletal pain [28,29,30]. In the latest Cochrane review of systematic reviews encompassing 30,700 participants from 206 studies, Derry et al. (2017) concluded that “There is good evidence that some formulations of topical diclofenac and ketoprofen are useful in acute pain conditions such as sprains or strains, with low (good) NNT values” [29]. Diclofenac 1% gel (Emulgel) showed high clinical relevance with an NNT (Number Needed to Treat) of 1.8. The earliest evidence for Tr14 application in treating ankle sprains was a randomized placebo-controlled clinical trial published in 1988 [31]. Another randomized clinical trial reporting Tr14 efficacy in treating soft tissue injuries was published later [32]. In the most recent randomized multicenter trial involving 449 patients from Spain, González de Vega et al. demonstrated that both topical Tr14 ointment and gel are non-inferior to diclofenac gel (1%) in managing pain and improving joint function, following a unilateral Grade 1 or 2 ankle sprain [23]. However, this trial included generally young, physically active patients (18–40 years of age) and did not include a placebo arm. The supporting evidence for Tr14’s effectiveness in musculoskeletal disorders was reviewed by van Haselen (2017) [33].

The scientific objective of the present trial was to overcome the mentioned limitations of the latest trial and to provide confirmatory evidence for the efficacy and safety of topical Tr14 gel in a much larger population, comparing it to a placebo and to an active comparator with a proven clinical relevance. Diclofenac (1% gel) was selected as a comparator with the largest clinical evidence base supporting anti-inflammatory agent use in acute musculoskeletal pain. Including a placebo arm ensured that a non-inferiority to an active comparator reflected the actual effect of Tr14 as a proposed inflammation-resolving agent.

Other objectives included studying changes over the course of the whole trial in the degree of pain on passive movement and at rest, effects on activities of daily living, requirement for rescue medication, and safety.

## 2. Methodology

### 2.1. Trial Design

This was a three-arm multicenter, prospective, randomized, double-blind trial. The investigators examined the effects of 7 days of topical treatment (Tr14 gel. vs. placebo gel vs. diclofenac gel) for acute ankle sprains (Grades 1 and 2). Subsequent follow-up was for a further 7 days (total trial duration 14 days; Table 1). The submitted trial protocol registered in Europe (EudraCT 2016-004792-50) was developed to comply with statutory requirements from the Bundesinstitut für Arzneimittel und Medizinprodukte (BfArM: German Federal Institute for Drugs and Medical Devices), and their advice regarding the optimal conduct of confirmatory trials. This was reinforced by a consensus of leading ortho-trauma specialists. All involved physicians and center-based research staff received training on practical aspects of the trial protocol, e.g., a standardized method of assessing pain on passive movement. Progress, quality, and protocol compliance during the trial were subject to regular centralized risk-based monitoring (RBM) by the external trial statistician and an independent external monitoring team. Following the ‘last patient last visit’, a meeting of involved and independent experts took place (Blind Data Review Meeting, BDRM). The BDRM members advised on which patients should be included in the final assessment groups.

### 2.2. Ethical Considerations

The clinical trial was conducted by a contract research organization (CRO: Advanced Medical Services, Mannheim, Germany) in accordance with the Declaration of Helsinki on Ethical Principles for Medical Research Involving Human Subjects, as adopted by the General Assembly of the World Medical Association (2013) [34]. The trial protocol complied with the International Council for Harmonisation Guideline for Good Clinical Practice (ICH-GCP) and all applicable national laws and regulations, including the archiving of essential documents [35]. Approval to conduct this trial was obtained from the central independent ethics committee (IEC) of the University of Kiel (reference number AZ A118/17) and the German Federal Institute for Drugs and Medical Devices (reference number 4042180). CONSORT guidelines for the conduct of randomized clinical trials and presentation of data were followed from the outset [36].

### 2.3. Participants

The main inclusion criteria were patients ≥18 years of age with a painful acute unilateral Grade 1 or Grade 2 sprain of the lateral ankle ≤24 h after trauma. Among the exclusion criteria were bilateral injuries, Grade 3 injuries, and trauma affecting the same ankle ≤6 months previously. More details are provided in Table 2. A fracture was excluded clinically and by x-ray examination where necessary [37].

The purpose of the trial was fully explained to potential participants. Elements discussed included the potential risks and benefits, gel application, putting on the elastic bandage, when/how to mobilize, and the use of rescue medication. This was supplemented by an information sheet. All participants signed the Informed Consent Form (ICF).

### 2.4. Interventions

Due to participation in this trial so soon after injury (≤24 h), further increases in tissue edema were to be expected. To reduce the risk of additional swelling and worsening pain, all patients received an elastic bandage as standard care. They were permitted to continue using the bandage for the duration of the trial if required. It was recommended that patients should use arm crutches and weight loading as tolerated for the duration of the trial, especially up to and including Day 4. Patients with a Grade 2 ankle sprain were supplied with a semi-rigid removable brace (orthosis) after re-examination from Day 7 onwards as required. The use of up to three elements of the RICE first-aid treatment was allowed prior to and after enrollment, while the use of all four RICE elements was prohibited after starting treatment and during the entire course of the trial.

#### Investigational Medicinal Products (IMP)

The composition of all IMPs is shown in Supplementary Table S1.

Test product: Tr14 gel (Traumeel^®^, Traumed, Heel GmbH, Baden-Baden) is a combination of 14 ingredients, mainly plant ethanol extracts. Three grams (3 g) were applied by gentle rubbing three times daily for 7 days to cover the affected area of the ankle.

Placebo: Corresponding placebo gel formulation, 3 g applied by gentle rubbing, three times daily for 7 days, to cover the area of the ankle.

Reference product: Diclofenac 1% gel (Heumann Pharma GmbH, Nuremberg, Germany), 3 g applied by gentle rubbing, three times daily for 7 days, to cover the affected area of the ankle.

Rescue medication: Paracetamol 500 mg tablets, to be ingested when necessary up to a maximum of 4 tablets per day (2000 mg/day) during the entire duration of the trial. Other pain relief medication was prohibited. Usage was recorded on the eCRF. This was compiled from information entered in the patient diary and the drug accountability form.

### 2.5. Blinding

Staff involved in the trial received comprehensive training. They were required to sign a form confirming that they understood the trial and agreed to adhere to the trial procedures. Research personnel, patients, sponsors, CROs, and other third-party vendors were blinded as to which treatment was being used. The local investigators kept the sealed treatment code (emergency) envelopes throughout the course of the trial and were not to break the code without a valid reason. No single case of unblinding was reported.

Blinding at each investigational site was achieved by only one person being assigned to handle the IMP, apply the soft support, and, in those with a Grade 2 ankle sprain, fit a semi-rigid brace on Day 7 as per protocol. This designated person administered the first dose of the IMP gel and trained the patient on how to use the dosing card (Supplementary Figure S1), apply the IMP, and fit the elastic bandage/semi-rigid brace. They were excluded from all other trial procedures related to the patient.

At each visit, the designated person removed the elastic bandage (and semi-rigid brace where appropriate) before the investigator arrived to examine the patient. Subsequently, the designated person reapplied the IMP and replaced the elastic bandage only after the investigator had left the room.

The designated person issued disposable dosing cards to be used for each IMP application, in order to standardize the amount of gel administered. Their camouflage design secured blinding (Supplementary Figure S1). The returned IMP was collected, registered, and returned to the sponsor for confirmative accountability and destruction.

### 2.6. Outcome Measures

The patient’s assessment of pain was performed by means of a 100 mm Visual Analog Scale (VAS) where ‘0’ was defined as ‘no pain’ and ‘100’ as the ’worst pain imaginable’. Efficacy was expressed in terms of changes in the calculated area under the curve (AUC) from baseline to a given measurement day. VAS measurements are a validated tool commonly used to assess subjective pain [38]. Calculation of AUC is a common standard in pain studies [39]. All AUC calculations from baseline to a given endpoint visit were based on the actual time of the included VAS measurements using the trapezoidal rule. Pain at rest was first assessed: patients were asked about their pain level scores on the VAS scale after 5 min of rest (pain at rest is a secondary efficacy assessment in this trial). Next, pain on passive movement was assessed (primary efficacy assessment): the investigator performed 10° dorsal and 30° passive plantar flexion on the injured ankle, and the patients again assessed their pain level scored using the VAS scale. The range of motion was controlled by using a goniometer. All investigators were trained at the investigator meeting to ensure a homogeneous assessment technique across all investigational sites. The date and time of the assessment were recorded. Individual pain assessments at each visit took place within +/−1 h of the original baseline assessment.

The primary efficacy endpoints were VAS scores for pain on passive movement, calculated as AUC from baseline to Days 4 and 7.

The original primary objective was to demonstrate the superior pain-reducing effects of Tr14 gel versus placebo gel in terms of AUC on passive movement at Day 4, with a secondary objective being non-inferiority to diclofenac. As defined in the protocol, the superiority, and non-inferiority hypotheses were pre-defined for confirmatory analysis in a priori order (see Statistical Analysis Plan (SAP)), with the test for superiority at Day 4 as hypothesis no. 1 (primary efficacy objective). To avoid a lack of assay sensitivity in the early stage after ankle joint injury, this was amended in the final SAP to examine pain on passive movement (AUC) at both Day 4 and Day 7 (primary outcome measures).

Secondary endpoints were VAS score changes for pain on passive movement and for pain at rest from baseline to all visits, calculated as AUC, percent-changes, and absolute values; time to 50% improvement in VAS for pain on passive movement and for pain at rest measured in days calculated from VAS assessments, performed at all patient visits using the percent change from baseline (recorded at Day 1). For the Foot and Ankle Ability Measure (FAAM) Activities of Daily Living (ADL) subscale, changes from baseline to all visits were calculated as absolute values and percentage changes. The FAAM is a validated questionnaire and was developed to meet the need for a self-reported evaluative instrument that comprehensively assesses the physical function of individuals with musculoskeletal disorders of the leg, foot, and ankle. The FAAM-ADL/S consists of an ADL (21-item scale) and a sports (S) subscale (8 items), asking study participants to rate their ability to perform specific tasks. In this clinical trial, FAAM-ADL was used (published on https://cdn-links.lww.com/permalink/jg9/a/jg9_2021_10_25_shah_21-00103_sdc2.pdf, accessed on 26 November 2023). Scoring of the FAAM-ADL subscale was performed according to the recommendations of Martin et al. (2005), with all scores transformed so that a lower value represents a higher level of physical function [40]. The amount of rescue medication at all visits was calculated as the number of ingested paracetamol tablets based on the information given in the patient diary and the drug accountability form for rescue medication during the trial.

Clinical safety was assessed by frequency and severity of adverse events (AEs), physical examination, and vital signs.

### 2.7. Randomization

Patients fulfilling the inclusion and exclusion criteria were randomly allocated to Tr14 gel, placebo gel, or diclofenac gel in a 2:1:1 randomization ratio. The investigators prepared a corresponding random code list using the random permuted block scheme. In accordance with the ICH Biostatistics Guideline, the block size was intentionally not given in the trial protocol. The sealed random code list and the sets of sealed envelopes were prepared using the validated program RANCODE in a validated working environment at IDV Data Analysis and Study Planning, Gauting, Germany.

Patients were randomized based on the 4-digit randomization number recorded on the investigational medicinal product (IMP). The next patient eligible for randomization was allocated to the lowest available randomization number at the site. IMP was provided to all sites in pre-defined shipment blocks. These contained an appropriate distribution of randomization codes belonging to the three treatment arms.

### 2.8. Sample Size

It was originally planned to enroll 624 patients. Estimation of sample size was based on the primary efficacy variable ‘pain on passive movement (AUC)’, as measured using a VAS from baseline to Day 4.

A two-sided test of equality for Tr14 gel and the comparator (placebo gel) at a level of 0.05 achieves a power of at least 90% for parametric first-line analysis based on an expected raw scale treatment difference of AUC 25 [mm x days] and a common standard deviation of AUC 75 [mm × days] for the response variables (re-expressed for nonparametric evaluation in terms of the Mann-Whitney statistic as MW = 0.6). This also holds for a second-line nonparametric analysis if the sample size is set to 291 patients for the Tr14 gel group and 146 patients for the placebo group.

Due to safety considerations in the event of a 1% AE incidence being encountered following treatment with the IMP at a probability level of 95%, and anticipating a drop-out rate of 4% with an allocation ratio of 2:1:1 (Tr14 gel: placebo gel: diclofenac gel), the statisticians calculated that this trial requires the inclusion of a total of 624 patients with an acute unilateral Grade 1 or Grade 2 ankle sprain.

During the recruitment phase site inspections by the German Local Authorities in 2019 (Gesundheitsamt Düsseldorf of German Federal State North Rhine Westphalia, NRW), it was recognized that there could have been a possible violation of the predefined ‘blinding firewall’ described in the protocol at some locations. The Principal Investigator, the Central Ethics Committee, and the Sponsor were informed. All sites were checked rigorously to detect any risk of unintentional unblinding, which, in the end, was not found. To exclude any risk of including these patients, the German regulatory authority (Federal Institute for Drugs and Medical Devices) recommended including only those cases in the final analysis where blinding could be 100% confirmed. The Sponsor, Trial Scientific Advisor, and biostatistical team increased the sample size to a total of 808 cases, which was documented in the amendment to the trial protocol on 3 February 2020. Their rationale was to maintain the planned power of the trial and ensure that the conclusions remained unbiased. The Federal Institute for Drugs and Medical Devices approved the amended protocol in March 2020, allowing the trial to continue.

### 2.9. Trial Procedures

The trial commenced with the recruitment of the first patient. The end of the trial was defined as the completion of the database lock.

After confirmation that the patient-matched the inclusion and exclusion criteria, the completion of the baseline trial procedures, and the signing of the ICF, participants were randomized to treatment with one of the three test products. The first application was during the screening/baseline visit (Day 1), which was then continued until the End of Treatment (EoT) visit on Day 7. After EoT, patients were followed up for another 7 days. Consequently, the entire duration of the trial for each patient was 14 days.

Patients kept an events diary. They were instructed to return their used gel tube and remaining paracetamol tablets to their treatment center. Compliance below 80% intake of IMP was considered a protocol violation, and the patient was then excluded from the Per Protocol (PP) set. The overview of trial procedures is shown in Table 1.

### 2.10. Statistical Analysis Plan (SAP)

Tr14 gel superiority versus placebo gel was evaluated using data from the Full Analysis Set (FAS) for all primary and secondary efficacy variables. The FAS was used for the Intention to Treat (ITT) analysis. The statistical analyses for the non-inferiority evaluation of Tr14 gel versus diclofenac gel were performed on the Per Protocol (PP) set for all efficacy variables.

Missing values for all efficacy parameters were imputed by the Last Observation Carried Forward (LOCF) approach.

The confirmatory analyses were performed using a parametric analysis of covariance (ANCOVA) for pain at baseline with the VAS as a covariate. Reported *p* values refer to the test differences between groups. Non-inferiority with respect to the primary endpoints was considered proven if the lower bound (LB) of the respective confidence interval for the difference estimator was LB ≥ −25 (pre-defined non-inferiority margin).

Pre-planned nonparametric sensitivity analyses were performed by means of the Wilcoxon-Mann-Whitney test (WMW), providing Mann-Whitney estimators (MW) as effect sizes with two-sided confidence intervals. As a benchmark for superiority, a MW of 0.64 was applied (referring to a standardized difference of 0.5 according to Cohen, which is regarded as a medium-sized difference). Other defined MW benchmarks were MW = 0.5 (equality), MW = 0.56 (small superiority), and MW = 0.71 (large superiority); pre-defined non-inferiority margin: MW = 0.4070.

Kaplan-Meier curves for time to 50% improvement in VAS scores for pain on passive movement and pain at rest were performed and tested for group differences by means of the Peto-Logrank test (time-to-event (log-rank) test).

All statistical tests were two-sided with a significance level of α = 0.05. Where appropriate, statistical tests were supported by presenting estimates and two-sided 95% confidence intervals for the respective treatment effects and differences between the treatment groups. These estimates and confidence intervals were based on the respective statistical models used for the analysis.

Confirmatory analyses for the primary outcome measures were defined prior to the unblinding in the Statistical Analysis Plan according to the following order:

1. AUC for pain on passive movement in VAS from baseline to Day 4 (test for superiority, Tr14 gel versus placebo gel, FAS). This was the primary efficacy objective of the trial.
2. AUC for pain on passive movement in VAS from baseline to Day 4 (test for non-inferiority, Tr14 gel versus diclofenac gel, PP). This was the secondary efficacy objective of the trial.
3. AUC for pain on passive movement in VAS from baseline to Day 7 (test for superiority, Tr14 gel versus placebo gel, FAS). Day 7 was implemented in the final SAP as an additional primary endpoint to avoid a lack of assay sensitivity in the early period following an ankle injury.
4. AUC for pain on passive movement in VAS from baseline to Day 7 (test for non-inferiority, Tr14 gel versus diclofenac gel, PP). As mentioned above, Day 7 was implemented in the final SAP as an additional primary endpoint to avoid a lack of assay sensitivity in the early period following an ankle injury.

If the preceding a priori-ordered test showed statistical significance, the subsequent hypothesis could then be tested individually in a confirmatory manner, according to the principle of priori-ordered hypotheses (for control of alpha using stepwise testing) [41]. In accordance with the ICH E9 Guidance, formal records have been kept of when the Statistical Analysis Plan was finalized, as well as when blinding was subsequently broken [42].

### 2.11. Safety

The safety population comprises all patients who received at least one dose of IMP. AEs were assessed at each visit in a descriptive manner. Where appropriate, the research team performed physical examinations and assessed vital signs.

## 3. Results

**Figure 1:**
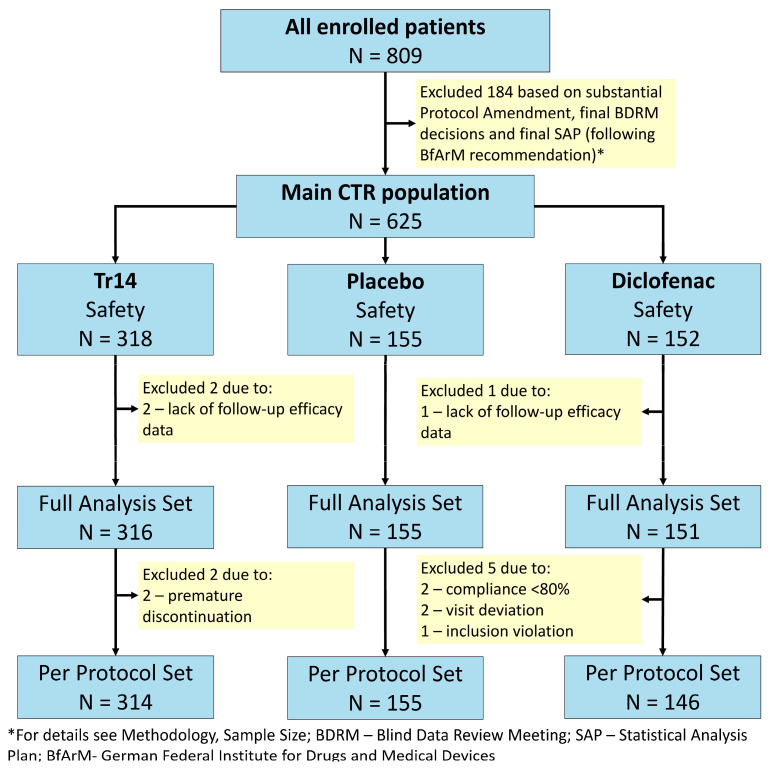
Participant flow.

A total of 809 patients (one more patient than planned) with Grade 1 or 2 ankle sprain were enrolled between 26 February 2018 and 18 November 2020 (Trial milestones Supplementary Table S2). Participating sites included 28 trauma centers, emergency units, sport medicine practices, and general practitioner offices in Germany. Participant flow is shown in Figure 1.

All randomized patients were treated with at least one dose of IMP. There were no safety exclusions, i.e., the safety dataset equals the randomized trial population (N = 625). Three patients were excluded from the safety data set due to a lack of follow-up data. Thus, the FAS population comprised 622 out of 625 patients in the safety data set: 316 patients in the Tr14 gel group (two exclusions), 151 patients in the diclofenac gel group (one exclusion), and 155 patients in the placebo gel group. With respect to the PP set (N = 615), 7 out of 622 patients (1.13%) were excluded from the FAS population (Tr14 gel: 2/316, diclofenac gel: 5/151, placebo gel: 0/155). Reasons for major protocol violations were premature discontinuation (N = 2), compliance <80% on Day 4 and/or Day 7 (N = 2), visit deviation (N = 2), and inclusion violation (N = 1). These limited protocol deviations are considered to have had negligible consequences for the patient populations.

### 3.1. Baseline Comparison

The groups were well comparable at baseline with respect to demographic-anamnestic criteria, supportive therapy, grade, time of injury, and efficacy criteria (Table 3). Body Mass Index (BMI) was statistically higher at baseline for the Tr14 gel group (mean BMI 26.2) versus the diclofenac gel group (mean BMI 24.8, MW = 0.5848, P_WMW_ = 0.0034). Analysis of Covariance (ANCOVA) on primary endpoints adjusted for BMI found that this imbalance was negligible and had no impact on the confirmatory results. Before adjustment, the AUC Day 4 mean difference (Tr14 vs. diclofenac) was 3.141 [95%CI −5.959–12.243] and *p* = 0.4812. The AUC Day 7 mean difference was 25.865 [95%CI 7.934–43.796] and *p* = 0.0034. After adjustment, the AUC Day 4 mean difference (Tr14 vs. diclofenac) was 5.175 [95%CI −5.458–15.808] and *p* = 0.3206. The AUC Day 7 mean difference was 27.675 [95%CI 7.717–47.633] and *p* = 0.0048.

### 3.2. Primary Efficacy Outcomes—From Baseline to Day 4 and Day 7 (End of Treatment)

The primary outcome measures in this trial were AUC for pain on passive movement in VAS from baseline to Day 4 and Day 7, combined with a test for superiority versus placebo and a test for non-inferiority versus diclofenac gel. The resulting hypotheses for confirmatory analysis were defined in the SAP in a specific order, with the test for superiority on Day 4 as hypothesis no. 1 (primary efficacy objective). The results are reported here in this pre-defined order.

**Figure 2:**
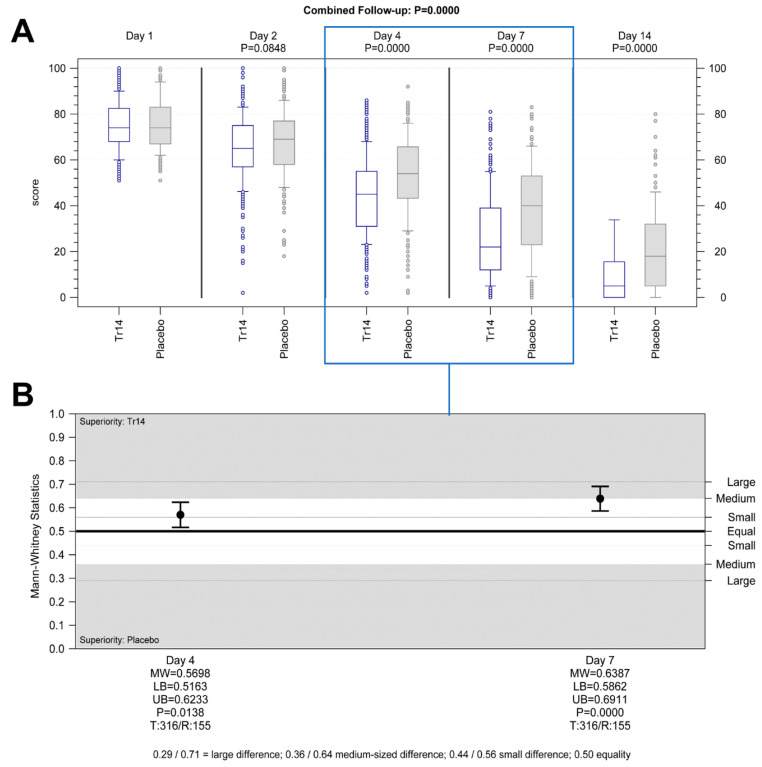
Primary efficacy outcomes: pain on passive movement Tr14 vs. placebo (**A**) absolute values, (**B**) AUC. (**A**) Boxplot shows absolute pain scores on VAS for passive movement for all visits (median, Percentile 10–90, LOCF) for Tr14 vs. Placebo (FAS). Primary endpoints (Day 4 and Day 7) are shown in the box. *p* values for combined follow-up represent non-parametric repeated measurements analysis. *p* values at single visits represent a two-sided Wilcoxon-Mann-Whitney U-test analysis. (**B**) The effect size for primary endpoints shows AUC for pain on passive movement on Days 4 and 7 (two-sided Wilcoxon-Mann-Whitney U-test, FAS, 95% Cl) for Tr14 vs. Placebo. Abbreviations: AUC = Area under the curve; VAS = Visual analog scale, LOCF = Last observation carried forward, FAS = Full Analysis Set, MW = Mann-Whitney estimator, LB = Lower bound of the two-sided confidence interval, UB = Upper bound of the two-sided confidence interval, P = *p*-value of Wilcoxon-Mann-Whitney test, T = Valid number of test group, R = Valid number of reference group.

**Hypothesis** **1.** Tr14 vs. placebo, Day 4, superiority analysis (FAS): Tr14 was significantly more effective in reducing pain after four days than placebo. The medians (min-max) for absolute VAS values on Day 4 were 45.0 (2.0–86.0) and 54.0 (2.0–92.0) (Figure 2A), and the median AUC scores were 187.88 (51.46–370.73) and 200.75 (86.08–374.43) for Tr14 and placebo respectively (Supplementary Table S3). The difference between the two treatment groups was statistically significant in the pre-defined confirmatory analysis (P_ANCOVA_ = 0.0205, two-sided; adjusted mean difference (MD_adj_) = 10.2; 95%CI 1.4 to 19.1; FAS population, Supplementary Table S4), as well as in the non-parametric sensitivity analysis (P_WMW_ = 0.0138, two-sided; MW = 0.5698; 95%CI 0.5163 to 0.6233, Figure 2B).

**Figure 3:**
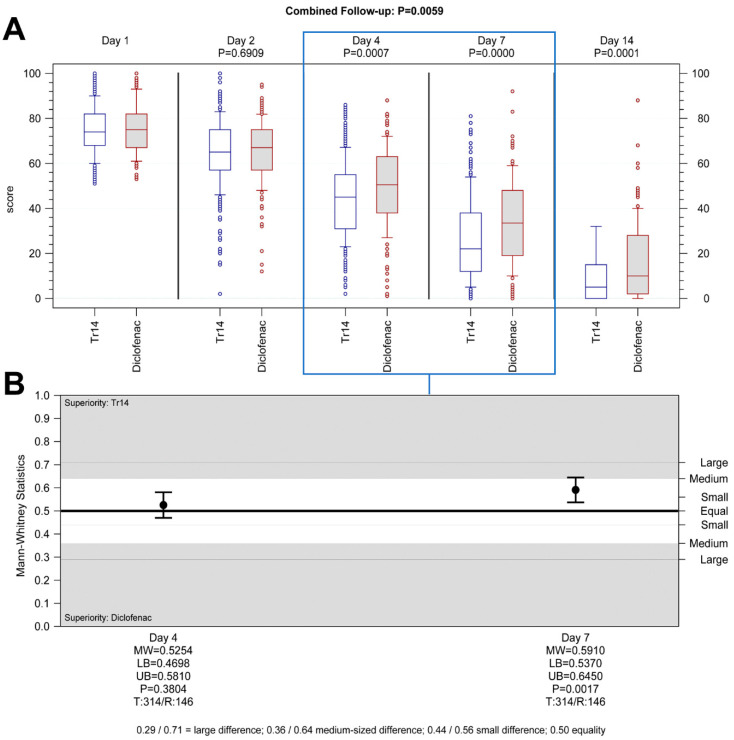
Primary efficacy outcomes: pain on passive movement Tr14 vs. diclofenac (**A**) absolute values, (**B**) AUC. (**A**) Boxplot shows absolute pain scores on VAS for passive movement for all visits (median, Percentile 10–90, LOCF) for Tr14 vs. diclofenac (PP). Primary endpoints (Day 4 and Day 7) are shown in the box. *p* values for combined follow-up represent non-parametric repeated measurements analysis. *p* values at single visits represent a two-sided Wilcoxon-Mann-Whitney U-test analysis. (**B**) The effect size for primary endpoints shows AUC for pain on passive movement on Days 4 and 7 (two-sided Wilcoxon-Mann-Whitney U-test, PP, 95% Cl) for Tr14 vs. Diclofenac. Abbreviations: AUC = Area under the curve; VAS = Visual analog scale, LOCF = Last observation carried forward, PP= Per Protocol set, MW = Mann-Whitney estimator, LB = Lower bound of the two-sided confidence interval, UB = Upper bound of the two-sided confidence interval, P = *p*-value of Wilcoxon-Mann-Whitney test, T = Valid number of test group, R = Valid number of reference group.

**Hypothesis** **2.** Tr14 vs. diclofenac, Day 4, non-inferiority analysis (PP): Tr14 was non-inferior to diclofenac in reducing pain after four days. The medians (min-max) for absolute VAS values on Day 4 were 45.5 (2.0–86.0) and 50.0 (1.0–88.0) (Figure 3A), and the median AUC scores were 187.50 (51.46–370.73) and 197.19 (76.74–366.54) for Tr14 and diclofenac respectively (Supplementary Table S3). Non-inferiority was proven in the confirmatory analysis by means of the confidence interval approach (pre-defined non-inferiority margin −25; MD_adj_ = 3.1; 95%CI −6.0 to 12.2; PP population, Supplementary Table S4), as well in the non-parametric sensitivity analysis (associated non-inferiority margin MW = 0.4070; MW = 0.5254; 95%CI 0.4698 to 0.5810, test for difference P_WMW_ = 0.3804, Figure 3B).

**Hypothesis** **3.** Tr14 vs. placebo, Day 7, superiority analysis (FAS): Tr14 was significantly more effective in reducing pain after seven days than placebo. The medians (min-max) for absolute VAS values on Day 7 were 22.0 (0.0–81.0) and 40.0 (0.0–83.0) (Figure 2A), and the median AUC scores were 294.14 (63.46–592.47) and 353.42 (101.28–620.07) for Tr14 and placebo respectively (Supplementary Table S3). The difference between the two treatment groups was again statistically significant (P_ANCOVA_ < 0.0001, two-sided; MD_adj_ 43.5; 95%CI 25.8 to 61.2; FAS population, Supplementary Table S4), as well as in the non-parametric sensitivity analysis (P_WMW_ < 0.0001, two-sided; MW 0.6387; 95%CI 0.5862 to 0.6911, Figure 2B).

**Hypothesis** **4.** Tr14 vs. diclofenac, Day 7, non-inferiority analysis (PP): Tr14 was both non-inferior and also superior to diclofenac in reducing pain after seven days. The medians (min-max) for absolute VAS values on Day 7 were 22.0 (0.0–81.0) and 33.5 (0.0–92.0) (Figure 3A), and the median AUC scores were 293.85 (63.46–592.47) and 327.93 (94.76–637.79) for Tr14 and diclofenac respectively (Supplementary Table S3). Non-inferiority was proven in the confirmatory analysis (pre-defined non-inferiority margin −25; MD_adj_ = 25.9; 95%CI 7.9 to 43.8; PP population, Supplementary Table S4), as well in the non-parametric sensitivity analysis (associated non-inferiority margin MW = 0.4070; MW = 0.5910; 95%CI 0.5370 to 0.6450, test for difference P_WMW =_ 0.0017, Figure 3B).

### 3.3. Secondary Efficacy Outcomes—From Baseline to All Visits

#### 3.3.1. Pain on Passive Movement

Tr14 gel significantly improved absolute VAS scores during the trial, compared to both placebo gel (combined follow-up nonparametric repeated measurement analysis *p* < 0.0001, Figure 2A) and diclofenac gel (combined follow-up nonparametric repeated measurement analysis *p* = 0.0059, Figure 3A, Supplementary Figure S2A,B) except for Day 2.

The percent-changes-from-baseline analysis showed, however, that VAS pain reduction by Tr14 vs. placebo was consistently significant at all visits, including Day 2 with small to large effect sizes (Day 2: median Tr14/placebo −11.11/−6.49; MW = 0.56, 95% CI 0.51–0.62, P_WMW_ = 0.02; Day 4: median −39.32/−25.93; MW = 0.65, 95% CI 0.60–0.71, P_WMW_ < 0.0001; Day 7: median −68.28/−45.68; MW = 0.67, 95% CI 0.62–0.72, P_WMW_ < 0.0001; Day 14: median −93.17/−77.27; MW = 0.69, 95% CI 0.64–0.74, P_WMW_ < 0.0001; Supplementary Figure S2C). In Tr14 vs. diclofenac comparison, Tr14 was superior to diclofenac except for Day 2 (Day 2 median Tr14/diclofenac −11.11/−8.28; MW= 0.53, 95% CI: 0.47–0.58, P_WMW_ = 0.319; Day 4: median −39.61/−28.17; MW = 0.60, 95% CI 0.55–0.66, P_WMW_ = 0.0003; Day 7: median −68.88/−55.66; MW = 0.62, 95% CI 0.57–0.67, P_WMW_ < 0.0001; Day 14: median −93.33/−84.98 MW = 0.61, 95% CI 0.56–0.67, P_WMW_ = 0001; Supplementary Figure S2D).

The descriptive statistics of the absolute values, AUC, and the percent-changes-from-baseline values are reported in Supplementary Table S3.

#### 3.3.2. Pain at Rest

**Figure 4:**
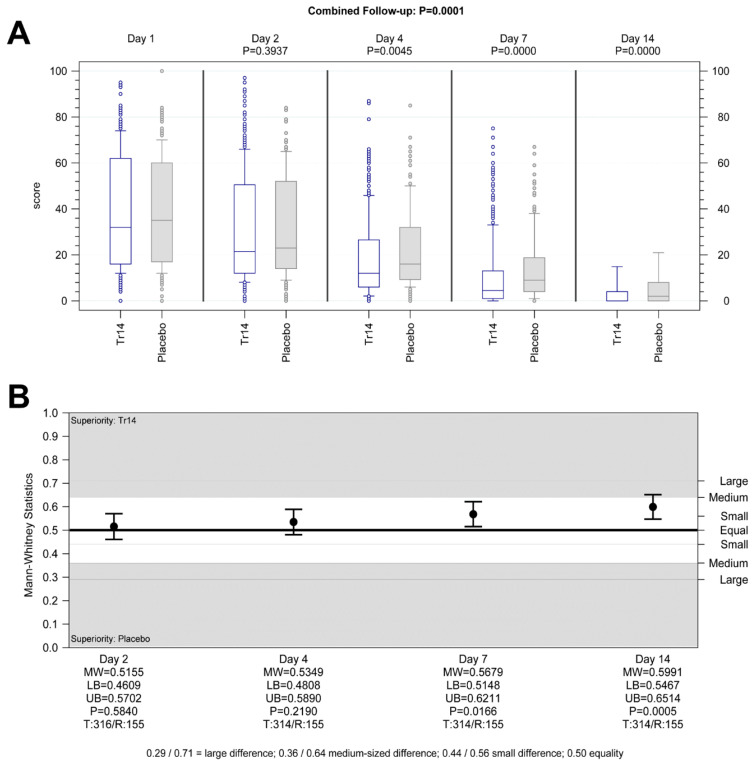
Secondary outcomes: pain at rest (all visits) Tr14 vs. placebo, (**A**) absolute values, (**B**) AUC. (**A**) Boxplot shows absolute pain scores on VAS for pain at rest for all visits (median, Percentile 10–90, LOCF) for Tr14 vs. Placebo (FAS). *p* values at single visits represent a two-sided Wilcoxon-Mann-Whitney U-test analysis. (**B**) The effect size for all visits shows AUC for pain at rest (two-sided Wilcoxon-Mann-Whitney U-test, FAS, 95% Cl) for Tr14 vs. Placebo. Abbreviations: AUC = Area under the curve; VAS = Visual analog scale, LOCF = Last observation carried forward, FAS = Full Analysis Set, MW = Mann-Whitney estimator, LB = Lower bound of the two-sided confidence interval, UB = Upper bound of the two-sided confidence interval, P = *p*-value of Wilcoxon-Mann-Whitney test, T = Valid number of test group, R = Valid number of reference group.

**Figure 5:**
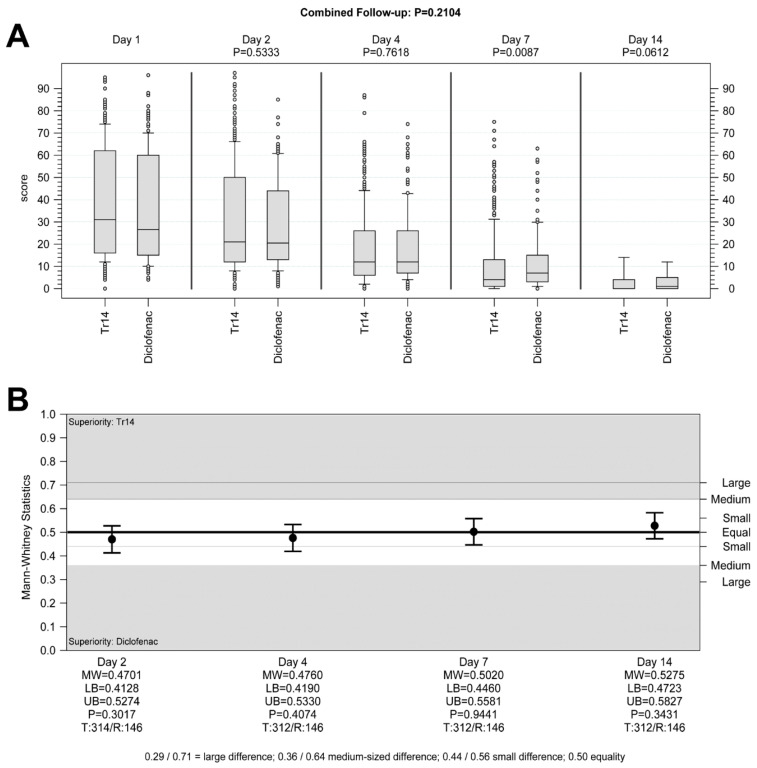
Secondary outcomes: pain at rest (all visits) Tr14 vs. diclofenac, (**A**) absolute values, (**B**) AUC. (**A**) Boxplot shows absolute pain scores on VAS at rest for all visits (median, Percentile 10–90, LOCF) for Tr14 vs. Diclofenac (PP). *p* values for combined follow-up represent non-parametric repeated measurements analysis. *p* values at single visits represent a two-sided Wilcoxon-Mann-Whitney U-test analysis. (**B**) Effect size shows AUC for pain at rest (two-sided Wilcoxon-Mann-Whitney U-test, PP, 95% Cl) for Tr14 vs. Diclofenac. Abbreviations: AUC = Area under the curve; VAS = Visual analog scale, LOCF = Last observation carried forward, PP = Per Protocol set, MW = Mann-Whitney estimator, LB = Lower bound of the two-sided confidence interval, UB = Upper bound of the two-sided confidence interval, P = *p*-value of Wilcoxon-Mann-Whitney test, T = Valid number of test group, R = Valid number of reference group.

Tr14 gel improved absolute VAS scores for pain at rest during the trial, compared to placebo gel (combined follow-up nonparametric repeated measurement analysis *p* = 0.0001, Figure 4A,B) except for Day 2. In comparison to diclofenac, Tr14 was non-inferior (combined follow-up nonparametric repeated measurement analysis *p* = 0.21, Figure 5A,B).

The percent-changes-from-baseline analysis showed consistently significant pain reduction by Tr14 vs. placebo at all visits, including Day 2, with small to medium effects (Day 2: median Tr14/placebo −15.29/−9.09; MW = 0.56, 95% CI 0.50–0.61, P_WMW_ = 0.04; Day 4: median −50.00/−36.86; MW = 0.62, 95% CI 0.56–0.67, P_WMW_ < 0.0001; Day 7: median −82.47/−63.49; MW = 0.65, 95% CI 0.60–0.70, P_WMW_ < 0.0001; Day 14: median −100.00/−92.16; MW = 0.64, 95% CI 0.59–0.69, P_WMW_ < 0001; Supplementary Figure S3). In the Tr14 vs. diclofenac comparison, Tr14 was superior to diclofenac except for Day 2 (Day 2: median Tr14/diclofenac −15.38/−12.05; MW= 0.53, 95% CI 0.48–0.59, P_WMW_ = 0.2514; Day 4: median −50.00/−42.86; MW = 0.56, 95% CI 0.51–0.62, P_WMW_ = 0.0290; Day 7: median −82.63/−73.51; MW = 0.61, 95% CI 0.56–0.66, P_WMW_ = 0.0001; Day 14: median −100.00/−97.43; MW = 0.56, 95% CI 0.50–0.61, P_WMW_ = 0.0343; Supplementary Figure S3).

The descriptive statistics of the absolute, AUC, and percent-changes-from-baseline values are reported in Supplementary Table S5.

#### 3.3.3. Foot and Ankle Ability Measure (FAAM)-Activities of Daily Living (ADL) Subscale

**Figure 6:**
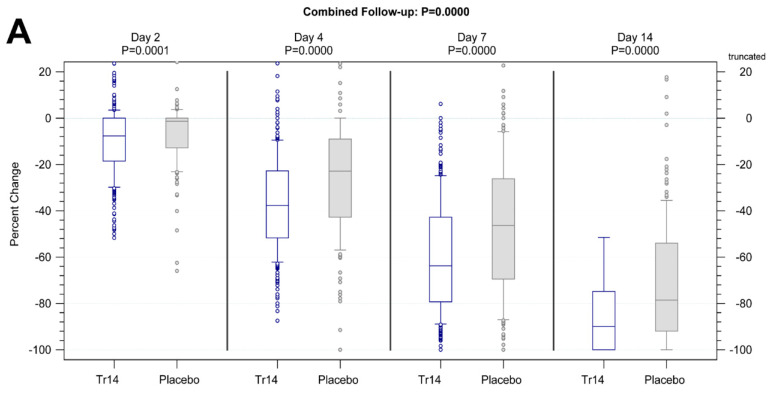

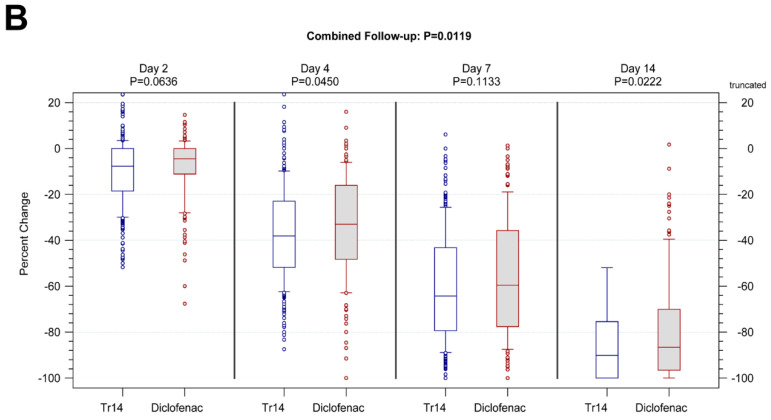
Secondary outcome: FAAM-ADL (all visits), percent change from baseline, (**A**) Tr14 vs. placebo (FAS), (**B**) Tr14 vs. diclofenac (PP). Boxplot shows percent-change-from-baseline of absolute FAAM-ADL subscale values (normalized of mean, percentage negative score FAS, LOCF, P10/P90) for all visits (median, percentile 10–90, LOCF). *p* values for combined follow-up represent non-parametric repeated measurement analysis. *p* values at single visits represent a two-sided Wilcoxon-Mann-Whitney U-test analysis. Abbreviations: FAAM-ADL = Foot and Ankle Ability Measure-Activities of Daily Living; LOCF = Last observation carried forward.

An examination of absolute values showed significant improvement in joint function following the application of Tr14 gel compared to placebo gel at all visits (combined follow-up nonparametric repeated measurement analysis *p* = 0.0004, Supplementary Figure S4A). The median values of Tr14/placebo were 47.42/46.43 (Day 2), 30.95/35.71 (Day 4), 17.11/26.19 (Day 7), and 4.76/9.52 (Day 14, Supplementary Table S6). Figure 6A shows the percent-change-from-baseline for Tr14 vs. placebo demonstrating medium effect size (Day 2: MW = 0.61, 95%CI 0.56–0.66, P_WMW_ = 0.0001; Day 4: MW = 0.65, 95%CI 0.60–0.70, P_WMW_ < 0.0001; Day 7: MW = 0.64, 95%CI 0.58–0.69, P_WMW_ < 0.0001; Day 14: MW = 0.64, 95%CI 0.59–0.70, P_WMW_ < 0.0001).

In absolute values, Tr14 gel was non-inferior to diclofenac gel at all visits (combined follow-up nonparametric repeated measurement analysis *p* = 0.2210; Supplementary Figure S4B). The median values for Tr14/diclofenac were −47.22/46.43 (Day 2), −30.95/32.14 (Day 4), 16.89/21.24 (Day 7), and 4.76/7.14 (Day 14, Supplementary Table S6). Figure 6B shows the percent-change-from-baseline for Tr14 vs. diclofenac, confirming non-inferiority results (effect size on Day 2: MW = 0.55, 95%CI 0.50–0.61, P_WMW_ = 0.0636; Day 4: MW = 0.56, 95%CI 0.50–0.61, P_WMW_ = 0.045; Day 7: MW = 0.55, 95%CI 0.49–0.60, P_WMW_ = 0.1133; Day 14: MW = 0.57, 95%CI 0.51–0.62, P_WMW_ = 0.0222), and showing Tr14 superiority at Days 4 and 14.

#### 3.3.4. Time to 50% Improvement in VAS Pain Scores

**Figure 7:**
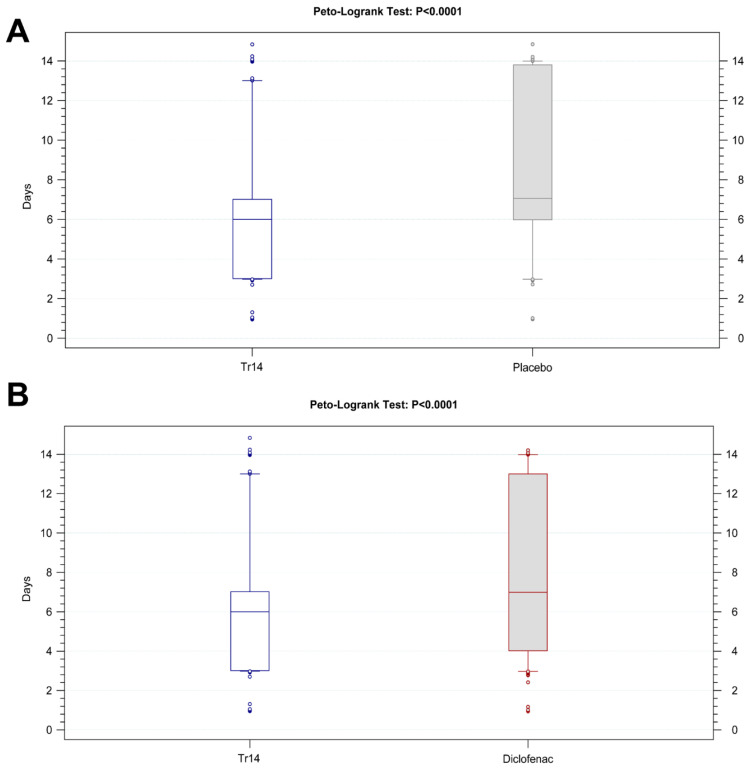
Secondary outcomes: Time (days) to 50% reduction of pain on passive movement, absolute values, (**A**) Tr14 vs. placebo (FAS), (**B**) Tr14 vs. diclofenac (PP). Boxplots show absolute values in days for time to 50% reduction of pain on passive movement for all visits (Median, Percentile 10–90, LOCF). Abbreviations: LOCF = Last observation carried forward, FAS = Full Analysis Set; PP = Per Protocol set.

For the pain on passive movement, the time to 50% improvement was (median) 6.0 days in the Tr14 gel group and 7.1 days in the placebo gel group (Figure 7A). The Kaplan-Meier analysis confirmed that a 1-day difference between the two medians is significant (*p* < 0.0001, Supplementary Figure S5). In the Tr14 versus diclofenac comparison, the time to 50% improvement was 6.0 days and 7.0 days, respectively. Similarly, a 1-day difference between the two medians was significant in favor of Tr14 gel (*p* < 0.0001, Figure 7B).

For pain at rest, the time to 50% improvement was 4.0 days in the Tr14 gel group and 6.0 days in the placebo gel group, and this 2-day difference between the two medians was significant (*p* = 0.0023, Supplementary Figure S6A). The Tr14 vs. diclofenac comparison showed a similar difference, which was not significant (4.0 days vs. 6.0 days, *p* = 0.2481, Supplementary Figure S6B).

For both outcomes, the trend for the median improvement time after Tr14 gel was shorter compared to both diclofenac gel and placebo gel.

#### 3.3.5. Rescue Medication

Requirements for rescue medication were small across all groups, with <15% of all patients taking paracetamol at any point during the whole trial. The mean of the total tablet consumption until the final visit was <1 tablet in all groups.

To evaluate the potential influence of rescue medication intake upon the VAS pain measurements, additional frequency counts were performed for the number of patients with rescue medication intake within the critical, predefined time window of 24 h, prior to the primary pain measurements. Interference rates were calculated for Days 4, 7, and 14, showing only negligible group differences with respect to interfering rescue medication. Regarding the comparison of Tr14 gel vs. diclofenac gel, the interference rate is ≤1% (Fisher exact test = 0.5548 for both Day 4 and Day 7). The highest (but still minimal) interference rate was observed in the placebo gel group, with 1.3% on Day 4. Additionally, we performed analyses of covariance (ANCOVA) with adjustment of the primary endpoints for the number of rescue medication tablets taken until the corresponding visit. The rescue-adjusted results confirmed the statistically significant superiority of Tr14 vs. placebo (rescue-adjusted/unadjusted: *p* = 0.0111/0.0205 for Day 4, *p* ≤ 0.0001/< 0.0001 for Day 7, and *p* ≤ 0.0001/< 0.0001 for Day 14). Regarding the comparison of Tr14 to diclofenac, there was no indication of the impact of the number of rescue medication tablets taken on the primary pain endpoint (*p* = 0.3535/0.4812 for Day 4, *p* = 0.0025/0.0034 for Day 7, and *p* < 0.0001/0.0001 for Day 14). Thus, the influence of rescue medication on the primary pain measurements may be regarded as negligible.

### 3.4. Safety

A total of 625 patients were randomized and received at least one dose of IMP (safety population). Adverse events (AEs) were assessed at each visit and recorded descriptively. In general, good safety and tolerability are encountered across all three treatment groups.

Altogether, 18 out of 625 patients (2.88%) suffered from 20 AEs. At least one AE was experienced by 9/318 patients (2.8%) in the Tr14 group, by 3/152 patients (1.97%) in the diclofenac group, and by 6/155 patients (3.9%) in the placebo group. One reported serious AE (unrelated humerus fracture in the diclofenac group), and all nonserious AEs are presented in Table 4. These were mostly mild in intensity and had resolved or were resolving by the trial’s end. No fatal AEs occurred in this trial. While the placebo had the highest AE rate in this trial, it is considered that the differences between the treatment groups occurred as a result of random variation (*p* = 0.7157).

The most common AE was headache (“migraine”, “headache”, “tension headache”), with five episodes occurring in three patients (0.8%), all in the Tr14 group. The headaches were of “mild” intensity, “unrelated”, and resolved on the same day; the “tension headache” was of “mild” intensity, “unrelated”, and resolved the next day; the “migraine” AE was of “moderate” intensity, “unrelated”, and also resolved the next day. The patient-based comparison to placebo with respect to this single AE category was statistically not significant (rate difference 0.0094, *p* = 0.5542, Fisher exact test). The second common AE was “dry skin” (four cases), with one case in the Tr14 gel group, two cases in the diclofenac gel group, and one case in the placebo gel group. Premature termination of IMP due to an AE was documented for 2/625 patients (0.32%; Tr14 gel:1; diclofenac gel:1). The majority of reported AEs were “mild”: 8/11 (73%) in the Tr14 group, 2/3 (67%) in the diclofenac group, and 5/6 (83%) in the placebo group. All of them “recovered” or were “recovering”, except one AE (abscess right elbow) in the Tr14 group, which was not IMP-related. Causality to investigational treatment was established for one AE in the Tr14 group (“dry skin”), two AEs in the diclofenac group (“dry skin”), and one AE in the placebo group (“dry skin”), all of “mild” intensity.

### 3.5. Clinical Relevance of Primary Outcomes—Post-Hoc Analysis

The primary goal of this trial was to confirm the efficacy of the Tr14 topical treatment by testing for superiority against the placebo, which was met. To assess possible clinical relevance, a post-hoc responder analysis was performed, applying alternative relevance benchmarks of the EMA pain Guideline [43]: “30 or 50 percent reduction in pain intensity compared to baseline”. The results are shown as rate differences (RD) comparing Tr14 vs. placebo (Supplementary Figure S7). On both primary outcome days (Day 4 and Day 7), these rate differences were highly significant (*p* < 0.0001), indicating clinically meaningful effects.

In comparison to diclofenac gel, Tr14 gel also showed statistically significant RDs for 50% pain reduction from baseline (Day 4: RD = 0.11 (95%CI 0.02 to 0.20), *p* < 0.01; Day 7: RD = 0.20 (95% CI 0.11 to 0.30), *p* < 0.0001).

## 4. Discussion

The availability of research data from high-quality trials seeking to optimize the management of acute ankle sprains remains limited and sometimes conflicting, as many widely used treatments lack EBM level 1 evidence [6]. Guidelines do exist but are often based on observation and opinion [4,44]. This three-arm, placebo-controlled randomized trial sought to generate high-quality evidence data by examining the effects of two widely used topical gels for Grades 1 and 2 ankle sprain injuries in adults, according to current guidelines for Good Clinical Practice. All groups were comparable at baseline, except for one variable—BMI in the PP population, which was slightly but significantly lower in the diclofenac group (BMI mean 26.2 for Tr14 group vs. 24.8 for diclofenac group, MW = 0.5848, P_WMW_ = 0.0034). In theory, the higher BMI in the Tr14 group could potentially have had an impact on the efficacy through higher dose requirements. However, analysis of covariance (ANCOVA) on primary endpoints showed that the BMI imbalance had only a negligible impact on the comparison: all BMI adjustments confirmed unadjusted results. In addition, we performed a subgroup analysis for the primary endpoints (Day 4 and Day 7) with dichotomization of BMI at the median of 25. The results with meta-analytic subgroup adjustment confirmed the unadjusted result (*p* = 0.47/0.4812 for Day 4, *p* = 0.003/0.0034 for Day 7). There was no indication of confounding effects of BMI with respect to the comparison of Tr14 and diclofenac. Age was well comparable across groups (median: 31 years in all three treatment groups). Analysis of covariance (ANCOVA) on primary endpoints showed that the age had no noticeable impact on efficacy results: all age adjustments confirmed the superiority of Tr14 vs. placebo (age-adjusted/unadjusted: *p* = 0.0243/0.0205 for Day 4, *p* < 0.0001/< 0.0001 for Day 7, and *p* < 0.0001/< 0.0001 for Day 14). To evaluate the question of whether treatment effects could be different in younger vs. older patients, we performed a subgroup analysis for the primary points in time by means of age dichotomization at the overall median of 31 years. As expected, the results with meta-analytic subgroup pooling confirmed the unadjusted result (*p* = 0.02/0.0205 for Day 4, *p* < 0.0001/< 0.0001 for Day 7). Formal tests for subgroup differences provided no indication for differences between younger and older patients (*p* > 0.5, all I^2^ = 0%). The same applied to the comparison of Tr14 vs. diclofenac (all tests for subgroup differences were with *p* > 0.5, all I^2^ = 0%).

All primary endpoints were met in the confirmatory analyses: Tr14 gel was superior to placebo gel and non-inferior to diclofenac gel at Days 4 and 7 in reducing the pain on passive movement. Considering that the baseline pain was in the upper moderate range (absolute mean >70 mm in all treatment groups on Day 1), the pain intensity drop of 20–23 mm on Day 4 can be considered as meaningful [45]. The change from baseline analysis showed a mean reduction of 30.6 (Tr14), 22.1 (placebo), and 25.5 (diclofenac), indicating a significant placebo effect, which is a well-described phenomenon in clinical trials investigating analgesia [46,47]. For example, in a recent clinical trial, topical placebo showed an adjusted VAS mean change of 11.32 mm from baseline to Day 4, while topical diclofenac reduced pain by 17.5 mm [48], which is a comparatively high placebo effect.

To assess the clinical relevance in a more meaningful way, we adopted the responder approach for the post-hoc analysis, applying alternative relevance benchmarks of the EMA pain Guideline [43]. The Guideline suggests that responder criteria should be defined for the primary efficacy measure according to a difference that is considered clinically meaningful to patients with the investigated pain condition. The minimally important clinical difference (MCID) is the smallest measurable change in outcome score that will make a difference to the patient. The IMMPACT group proposed defining the MCID as a 30% or greater improvement in self-reported pain and function, and a 50% and greater improvement was suggested to be a substantial change in pain [49]. The MCID may vary depending on the patients and the clinical context, and the EMA Guideline suggests similar benchmarks “30 or 50 percent reduction in pain intensity compared to baseline”, which we used for our post-hoc analysis. It showed that within these benchmarks, Tr14 had statistically significant rate differences compared to placebo and diclofenac on both primary outcome days, which is a good indication for clinically relevant effects of Tr14 in patients with acute ankle sprain. The TRAUMED trial adds more confidence about the pain-reducing efficacy of Tr14 gel, which was demonstrated previously.

A previous diclofenac-controlled clinical trial of Tr14 ointment/gel was conducted in 449 individuals with Grade 1 or Grade 2 acute ankle sprain [23]. Participants were described as physically active and generally younger than in the current trial, but there was no placebo group. The baseline medians of the pain VAS scale were lying between 52.6 mm and 55.7 mm (minimum 29.9 mm, maximum 94.8 mm), i.e., participants were in less pain than in the current trial. All groups showed strong pain decreases with a final median of 0 mm (total pain relief) at a longer follow-up period (6 weeks). At the primary endpoint of Day 7, the median was 21.6 (Tr14 ointment), 16.0 (Tr14 gel), and 17.5 (diclofenac gel), demonstrating non-inferiority of Tr14 compared to diclofenac. The TRAUMED trial confirms efficacy by demonstrating non-inferiority to diclofenac gel and additionally shows superiority to placebo.

The secondary outcomes were consistent with the primary analyses. Tr14 improved pain at rest compared to placebo, although to a lesser absolute degree than pain on passive movement and was non-inferior to diclofenac. The contradicting effects of NSAIDs for the pain at rest are well known [6]. The difference of Tr14 absolute effects between movement-evoked pain and pain at rest might well be explained by the substantially lower baseline values of pain at rest, introducing early floor effects (pain at rest level was at baseline less than half of pain on passive movement level) [50]. We assume similar differences between both pain outcomes in this trial are applicable. Function improvement by Tr14 (FAAM ADL, superiority to placebo, and non-inferiority to diclofenac) of the impaired ankle was consistently demonstrated throughout all visits. Our data also indicates that pain resolution is hastened by at least 1 day following the application of Tr14 gel (time to 50% improvement). Faster return to sports (RTS) could bring value to athletes and their teams. This is an important consideration for members of football, soccer, basketball, and volleyball clubs, amongst whom ankle injury rates are high. If a professional player can return to fitness in time for a crucial match, this could influence their team’s performance, their success at the end of the season, and their future club revenues [51,52]. Studies in athletes show that RTS, 8 days after injury, is common. However, many patients return with non-resolved pain, impaired function, and continued disability [53]. Recently, an Ankle-GO score was developed to help in the decision-making regarding RTS after a lateral ankle sprain, which includes FAAM-ADL but does not consider pain relief [54]. Other groups suggested the PAASS framework, which includes a pain domain but does not consider FAAM [55]. This coincides with the opinions of Gaddi et al. (2022) regarding a lack of consensus on which factors influence RTS [4]. We suggest that both the FAAM-ADL data and time to 50% improvement of pain are relevant for RTS and, in relation to the present results, demonstrate the benefits of Tr14. While the TRAUMED trial did not focus on athletes, de Vega et al. reported after a 42-day follow-up, a median RTS of 14 days for Tr14 and 19 days for diclofenac (the difference was not significant) [23], an indication that for many RTS after 8 days could be premature.

The safety profiles of all treatments were comparably strong; AEs across all groups were minimal (2.88%), consisting mainly of headache and local irritation, and were quick to resolve. To interpret the safety of this trial in the context of previous knowledge, we have looked at the pharmacovigilance data on Tr14 gel between 1 January 2008 and 1 January 2016. It showed nine non-serious adverse drug reactions (ADRs), assessed with “possible” or “probable” causal relationship to the treatment. Two of the cases reporting skin reactions were received from a clinical trial (TAASS trial/EudraCT number: 2008-007939-4). One case reported eye hypersensitivity symptoms and can be considered an isolated case. The other cases related to hypersensitivity skin conditions were already listed in the respective labels (allergic reactions). According to the trial Sponsor, the incidence ratio for the non-serious ADRs was 7 cases per 2.047.579 packages sold with an event rate of 1:292.511, which makes it very rare (very rare is defined as less than 1:10.000 or 0.01%). In the present trial, only four (4) out of 20 AEs were judged as ADRs (having a causal relationship to IMP): one experienced by a Tr14-treated patient, one by a placebo-treated patient, and two by patients from the diclofenac group. All four ADRs were dry skin (at the treated area). No AEs indicative of Tr14 gel overdose have been reported. We should note that the most common AE was headache, reported only in the Tr14 group (5 AEs in three patients). In the TAASS trial, headache was also the most common AE (43 adverse events were reported in total by 31/447 [6.9%] patients—none of them were serious). In both trials, investigators assessed headaches as non-related to the treatment. In conclusion, Tr14 may be regarded as a safe treatment for patients with moderate to severe ankle sprain, with no serious AEs, and all reported AEs resolved on the same or next day. Observed group differences compared to placebo are easily explained by chance (*p* > 0.5).

This clinical trial was designed to generate widely applicable results. It was developed in close cooperation with the German regulatory authority (Federal Institute for Drugs and Medical Devices), supervised by central and local authorities, and monitored by the statistical vendor using a centralized risk-based approach. The clear findings confirm the benefit and safety of topical treatment following an acute ankle sprain. Furthermore, Tr14 gel was found to be non-inferior to the global market leader, i.e., diclofenac gel. Our data show that Tr14 gel provides superior pain control compared to placebo and a faster return to normal activities than its active comparator. This aspect is not addressed in most RCTs but is important in sports medicine, especially in a professional sports environment. It should be noted that there was a higher paracetamol usage in the Tr14 group. However, because of the small number of tablets involved (less than 15% of patients required rescue medication at any time), statistical analysis demonstrated that the influence of paracetamol consumption on primary outcomes was negligible. Overall, this trial adds more evidence for considering Tr14 in the management of ankle sprains, especially in the context of other pharmaceutical treatment modalities, which did not result in a quicker time to return to activity [6].

Ankle sprains are the most prevalent musculoskeletal injury in physically active individuals [56]. They are not only painful and disabling but are also typically associated with significant societal and economic consequences [2]. Cooke et al. [57] reported the short-term direct costs in the UK for a patient attending the emergency department with a sprained ankle to be £940, while data from the Netherlands estimate the costs to be €823 [58]. By comparison, Knowles et al., in the USA, calculated both short-term and indirect costs as well as estimated the monetary value of lost health, resulting in a figure of $11,925 per ankle injury [59]. This means that if there are approximately 628,000 ankle sprains occurring annually in the USA, this will result in a financial burden of more than $6 billion. This is likely to be an underestimate if <50% of patients seek formal medical care. For the individual and employer, indirect costs include loss of paid work and productivity. Cooke et al., for example, estimated a loss of 6.9 days of paid work due to an ankle sprain treated by standard means [57].

A further consideration is optimizing homeostasis. While oral NSAIDs are associated with a range of side effects in a minority of individuals, topical administration is generally well tolerated [60]. Evidence shows that in acute pain, systemic or local adverse event rates with topical NSAIDs (4.3%) were no greater than with a topical placebo (4.6%) (42 studies, 6740 participants, high-quality evidence) [29]. Hence, its widespread usage and ‘over the counter’ promotion. AE rates in this trial were comparably low, not different from the placebo group. So why seek to challenge its perceived supremacy? The reason is that mounting evidence points to the anti-inflammatory properties of NSAIDs as interfering with normal tissue healing and promoting chronicity [24]. Some evidence-based clinical guidelines also mention that the use of NSAIDs may delay the natural healing process as the inflammation suppressed by NSAIDs is a necessary component of tissue recovery [6]. By comparison, agents such as Tr14, which initially encourage local neutrophil activity and subsequent clearance, may aid homeostasis. It is suggested that treatments that support the physiological reaction to inflammation are likely to be more effective than medicines that have a suppressive effect. Stimulating inflammation-resolution pathways is probably one of the key mechanisms of action of Tr14 [21,22]. The potential therapeutic value of influencing soft tissue healing on a cellular level has been demonstrated elsewhere [61].

Total pain relief at Day 7 (100.0%) was reached in 8.5% of the patients in the Tr14 ointment group, in 5.0% of the patients in the Tr14 gel group, and in 5.9% of the patients in the diclofenac gel group. The baseline medians of the FAAM-ADL score were lying between 51.2 and 56.0 (minimum 2.4, maximum 98.3). All groups showed a strong increase of FAAM-ADL with a final median of 100 (best score) after 6 weeks. At the primary endpoint, Day 7, the median was 81.0 (Tr14 ointment), 85.1 (Tr14 gel), and 79.8 (diclofenac gel). Thus, the best median score was observed in the Tr14 gel group. As in the current trial, the overall safety for all IMP was considered very good, with 43 diverse AEs among 447 patients receiving Tr14 ointment/gel or a placebo (6.9%), though the AE profile was a little different with a preponderance of headaches [23].

### Limitations

One limitation of this trial is the short duration. We do not know what happened to participants after 14 days. It may be that some suffer further injury or develop chronic ankle problems. If claims are to be made regarding superior healing and avoidance of chronicity after administration of Tr14, then longer-term, well-formulated data is needed. Another consideration is whether the use of an elastic bandage, together with Tr14 gel, might have influenced efficacy by modifying the biomechanics of ankle stability. To address this, a further follow-up stage could have been incorporated, but data collection is likely to be affected by a high dropout rate among the many young participants who are generally believed to make a good recovery. Consequently, it is not possible to recommend the most effective treatment protocol based solely on the present results. This needs to be addressed in further studies looking at dose optimization, application intervals, treatment duration, and usage of Tr14 gel as part of multimodal treatment.

## 5. Conclusions

Tr14 gel is a beneficial and well-tolerated medication for the early treatment of patients with acute ankle sprains. This trial examined two primary outcome measures (VAS AUC at Day 4 and Day 7) and two primary group comparisons (Tr14 superiority to placebo and Tr14 non-inferiority to diclofenac), leading to four a priori-defined hypotheses. These were all confirmed. Pain control following Tr14 was found to be superior to placebo on Day 4 and Day 7, non-inferior to diclofenac on Day 4, and superior to diclofenac on Day 7. Tr14 was also superior to both diclofenac and placebo on Day 14. The FAAM-ADL subscale demonstrated Tr14 to be superior to placebo at all visits and non-inferior to diclofenac. Additionally, nonparametric sensitivity analysis found Tr14 to be superior to diclofenac on Day 4. Time to 50% improvement was shorter compared to placebo and diclofenac. AEs were uncommon and mild across all groups. While further research is needed around dosage, duration, and the exploration of long-term effects of topical treatment, when presented with a patient suffering a mild ankle sprain (Grades 1–2), early application of Tr14 is a safe and effective option.

## Figures and Tables

**Table 1:** Summary of timelines and interventions.

	Visit 1 Initial Screening/Baseline	Visit 2	Visit 3	Visit 4	Visit 5 Final
	Day 1 Randomization and Start of Treatment	Day 2	Day 4 Primary Outcome	Day 7 Primary Outcome	Day 14
History and examination	X				
Confirm injury ≤24-h	X				
Confirm Grade 1 or 2 injury	X		X		
Physical examination	X				X
Confirm inclusion and exclusion criteria	X				
Obtain informed consent	X				
Exclude pregnancy (if applicable)	X			X	
Application of IMP gel	X	X	X	X	
Application of soft support (elastic bandage)	X	X	X	As required	As required
Rescue medication dispensed	X			As required	As required
Assessment VASPM	X	X	X	X	X
Assessment VASPR	X	X	X	X	X
FAAM-ADL	X	X	X	X	X
Diary reviewed		X	X	X	X
Rescue medication review		X	X	X	X
Record AEs		X	X	X	X
Application of semi-rigid brace for Grade 2				X	X

IMP: Investigational medicinal product; VASPM: Visual analog scale pain on passive movement; VASPR: Visual analog scale pain at rest; FAAM-ADL = Foot and Ankle Ability Measure—Activities of Daily Living; AE: Adverse event.

**Table 2:** Inclusion and exclusion criteria.

Inclusion Criteria
Acute unilateral Grade 1 or Grade 2 sprain of the lateral ankle
≥18 years of age
Legally competent male or female outpatient
Injury occurred within previous 24 h before first treatment expected
After 5 min of rest, pain on passive movement by investigator measured by Visual Analog Scale (VAS) ≥50 mm
Not pregnant or breastfeeding
Signed Informed Consent
**Exclusion Criteria**
Similar injury affecting the same joint within the past 6 months
Bilateral ankle injury
Grade 3 ankle sprain
Fracture of the ankle
Chronic joint disorders
Disorders that may lead to joint edema for other reasons, other than ankle sprain
Diagnosis requiring bed rest, hospitalization, surgery, or use of any cast during the planned treatment period
Debilitating acute or chronic illness
Use of systemic and/or topical corticosteroids in the previous 8 weeks, any analgesics in the previous 24 h before Screening Visit, or 48 h in the case of long-acting NSAID, cyclooxygenase type 2 (COX-2) specific inhibitors, or tramadol and other opioids. Low dose acetylsalicylic acid (70–100 mg per day) for anti-thrombotic therapy was permitted if doses were stable for the month prior to Screening Visit and planned to be stable during the entire trial
History of sensitivity to any component of the trial drugs
Unwilling or unable to comply with all the requirements of the study protocol
Concurrent injury to proximal structures in ipsilateral lower extremity
History of ligament avulsion, fracture, or surgery to the affected lower extremity
Presence of infections and/or skin diseases at the investigational treatment site (including psoriasis)
Any previous treatments of the injured ankle, whether topical or systemic, were prohibited except RICE (simultaneous combination of all 4 elements Rest, Ice, Compression and Elevation, which was restricted to be used until starting treatment with the IMP)
Participation in any clinical study within the past 4 weeks
Any relationship of dependence with the sponsor or with the investigator

**Table 3:** Baseline characteristics.

Baseline Characteristics	Tr14 Gel N = 316	Placebo Gel N = 155	Diclofenac Gel N = 151
**Age (Years) Mean (SD, Range)**	35.2 (14.11, 18–78)	36.0 (13.66, 18–76)	34.7 (13.53, 18–75)
Age range 18–64 years	305	152	147
Age range 65–78 years	11	3	4
**Gender, number (%)**			
Female	156 (49.4%)	68 (43.9%)	64 (42.4%)
Male	160 (50.6%)	87 (56.1%)	87 (57.6%)
**Ethnic origin, number (%)**			
Caucasian	305 (96.5%)	152 (98.1%)	149 (98.7%)
Black	3 (1.0%)	2 (1.3%)	0
Asian	1 (0.3%)	0	0
Other	7 (2.2%)	1 (0.6%)	2 (1.3%)
**Injury Grading, number (%)**			
Grade 1	216 (68.4%)	103 (66.5%)	109 (72.2%)
Grade 2	100 (31.6%)	52 (33.5%)	42 (27.8%)
**Body Mass Index (BMI, kg/m^2^) Mean (SD)**	26.2 (4.80)	26.2 (4.63)	24.7 (4.08)
**Supportive Therapy, number (%)**			
Elastic bandage	316 (100.0%)	155 (100.0%)	151 (100.0%)
Arm crutches	78 (24.7%)	51 (32.9%)	40 (26.5%)
Foot cooled	170 (53.8%)	77 (49.7%)	76 (50.7%)
Extra rest	165 (52.2%)	74 (47.7%)	71 (47.3%)
Foot cooled with ice	25 (7.9%)	11 (7.1%)	16 (10.7%)
Compression wrap	6 (1.9%)	1 (0.7%)	7 (4.7%)
Foot elevated	180 (57.0%)	82 (52.9%)	77 (51.3%)
**Efficacy Outcomes**			
VASPM Mean (SD, Range)	74.9 (10.97, 51–100)	75.5 (11.36, 51–100)	74.9 (11.41, 43–100)
VASRS Mean (SD, Range)	39.4 (24.65, 0–95)	39.7 (23.88, 0–100)	37.0 (23.87, 4–96)
FAAM-ADL Mean (SD, Range)	51.4 (17.36, 0–95)	49.7 (18.01, 0–98)	50.6 (18.59, 0–98)

VASPM: Visual analog scale pain on passive movement; VASPR: Visual analog scale pain at rest; FAAM-ADL = Foot and Ankle Ability Measure—Activities of Daily Living.

**Table 4:** Serious and non-serious Adverse Events (AEs).

Serious AEs	Tr14 n = 318	Placebo n = 155	Diclofenac n = 152
Patients affected/exposed	0/318	0/155	1/152 (0.66%) *
Deaths (all causes)	0	0	0
**Nonserious AEs**			
Patients affected/exposed	9/318 (2.83%)	6/155 (3.87%)	2/152 (1.32%)
*Injury related*			
Associate fibula fracture (Weber A)	1/318 (0.31%)	0/155	0/152
Forefoot distortion	1/318 (0.31%)	0/155	0/152
Sacroiliac sprain	0/318	1/155	0/152
*Nervous system disorders*			
Migraine	1/318 (0.31%)	0/155	0/152
Headache	3/318 (0.94%)	0/155	0/152
Tension headache	1/318 (0.31%)	0/155	0/152
*Administration site conditions*			
Application site burning after previous mosquito bite	0/318	1/155 (0.65%)	0/152
Dry skin	0/318	1/155 (0.65%)	0/152
Dry skin over treated (skin) area	1/318 (0.31%)	0/155	2/152 (1.32%)
*Musculoskeletal and connective tissue disorders*			
Neck pain	1/318 (0.31%)	0/155	0/152
Knee pain left	0/318	1/155 (0.65%)	0/152
*Infections*			
Acute nasopharyngitis	0/318	1/155 (0.65%)	0/152
Common cold	1/318 (0.31%)	1/155 (0.65%)	0/152
Abscess right elbow	1/318 (0.31%)	0/155	0/152

* Humerus fracture unrelated to Investigational Medicinal Product; AE: Adverse Event.

## Data Availability

The datasets supporting the conclusions of this article are included with this article and the additional supplementary files. Additional datasets generated during the current study are not publicly available due to ongoing analysis. Subject to commercial considerations, they will subsequently be available from the trial sponsor for scientific purposes upon receipt of a reasonable request.

## Supplementary Materials

The following supporting information can be downloaded at: https://www.mdpi.com/article/10.3390/jcm13030841/s1, Supplementary Table S1: Composition of the investigational medicinal products (IMP). Supplementary Table S2: Trial milestones. Supplementary Table S3: Median values of AUC, absolute scores, and percent-change-from-baseline for VAS scores for pain on passive movement (VASPM), LOCF. Supplementary Table S4: Parametric ANCOVA analysis VAS AUC scores for pain on passive movement (AUCPM) for comparisons Tr14 vs. placebo (FAS) and Tr14 vs. diclofenac (PP). Supplementary Table S5: Median values of AUC, absolute scores, and percent-change-from-baseline for VAS scores on pain at rest (VASRS), LOCF. Supplementary Table S6: Median values of absolute scores and percent-change-from-baseline for FAAM-ADL scores, LOCF. Supplementary Figure S1: Disposable Dosing Card. Supplementary Figure S2: Pain on passive movement (VASPM) at all visits. Supplementary Figure S3: Pain at rest (VASRS) at all visits. Supplementary Figure S4: Foot and Ankle Ability Measure—Activities of Daily Living (FAAM-ADL) at all visits. Supplementary Figure S5: Kaplan-Meier function for a time in days to a 50% reduction in absolute VAS scores for pain on passive movement (VASPM) and pain at rest (VASRS). Supplementary Figure S6: Time in days to 50% reduction of pain at rest in absolute VAS score. Supplementary Figure S7: Post-hoc responder analysis for 30% and 50% pain reduction for Tr14 gel compared to placebo gel.

## References

[1] Herzog M.M., Kerr Z.Y., Marshall S.W., Wikstrom E.A. (2019). Epidemiology of Ankle Sprains and Chronic Ankle Instability. J. Athl. Train..

[2] Gribble P.A., Bleakley C.M., Caulfield B.M., Docherty C.L., Fourchet F., Fong D.T.-P., Hertel J., Hiller C.E., Kaminski T.W., McKeon P.O. (2016). Evidence review for the 2016 International Ankle Consortium consensus statement on the prevalence, impact and long-term consequences of lateral ankle sprains. Br. J. Sports Med..

[3] Halabchi F., Hassabi M. (2020). Acute ankle sprain in athletes: Clinical aspects and algorithmic approach. World J. Orthop..

[4] Gaddi D., Mosca A., Piatti M., Munegato D., Catalano M., Di Lorenzo G., Turati M., Zanchi N., Piscitelli D., Chui K. (2022). Acute Ankle Sprain Management: An Umbrella Review of Systematic Reviews. Front. Med..

[5] Hubbard T.J., Cordova M. (2009). Mechanical instability after an acute lateral ankle sprain. Arch. Phys. Med. Rehabil..

[6] Vuurberg G., Hoorntje A., Wink L.M., Van Der Doelen B.F.W., Van Den Bekerom M.P., Dekker R., Van Dijk C.N., Krips R., Loogman M.C.M., Ridderikhof M.L. (2018). Diagnosis, treatment and prevention of ankle sprains: Update of an evidence-based clinical guideline. Br. J. Sports Med..

[7] Kerkhoffs G.M., Van Den Bekerom M., Elders L.A.M., Van Beek P.A., Hullegie W.A.M., Bloemers G.M.F.M., De Heus E.M., Loogman M.C.M., Rosenbrand K.C.J.G.M., Kuipers T. (2012). Diagnosis, treatment and prevention of ankle sprains: An evidence-based clinical guideline. Br. J. Sports Med..

[8] Struijs P.A.A., Kerkhoffs G.M.M.J. (2015). Ankle sprain: The effects of non-steroidal anti-inflammatory drugs. BMJ Clin. Evid..

[9] Curtis E., Fuggle N., Shaw S., Spooner L., Ntani G., Parsons C., Corp N., Honvo G., Baird J., Maggi S. (2019). Safety of Cyclooxygenase-2 Inhibitors in Osteoarthritis: Outcomes of a Systematic Review and Meta-Analysis. Drugs Aging.

[10] Ussai S., Miceli L., Pisa F.E., Bednarova R., Giordano A., Della Rocca G., Petelin R. (2015). Impact of potential inappropriate NSAIDs use in chronic pain. Drug Des. Devel Ther..

[11] Wöhrl S. (2018). NSAID hypersensitivity—Recommendations for diagnostic work up and patient management. Allergo J. Int..

[12] Parisien M., Lima L.V., Dagostino C., El-Hachem N., Drury G.L., Grant A.V., Huising J., Verma V., Meloto C.B., Silva J.R. (2022). Acute inflammatory response via neutrophil activation protects against the development of chronic pain. Sci. Transl. Med..

[13] van Walsem A., Pandhi S., Nixon R.M., Guyot P., Karabis A., Moore R.A. (2015). Relative benefit-risk comparing diclofenac to other traditional non-steroidal anti-inflammatory drugs and cyclooxygenase-2 inhibitors in patients with osteoarthritis or rheumatoid arthritis: A network meta-analysis. Arthritis Res. Ther..

[14] Taylor R.S., Fotopoulos G., Maibach H. (2011). Safety profile of topical diclofenac: A meta-analysis of blinded, randomized, controlled trials in musculoskeletal conditions. Curr. Med. Res. Opin..

[15] Sathishkumar P., Meena R.A.A., Palanisami T., Ashokkumar V., Palvannan T., Gu F.L. (2020). Occurrence, interactive effects and ecological risk of diclofenac in environmental compartments and biota—A review. Sci. Total Environ..

[16] Serhan C.N. (2014). Pro-resolving lipid mediators are leads for resolution physiology. Nature.

[17] Markworth J.F., Vella L., Lingard B.S., Tull D.L., Rupasinghe T.W., Sinclair A.J., Maddipati K.R., Cameron-Smith D. (2013). Human inflammatory and resolving lipid mediator responses to resistance exercise and ibuprofen treatment. Am. J. Physiol.-Regul. Integr. Comp. Physiol..

[18] Serhan C.N., Libreros S., Nshimiyimana R. (2022). E-series resolvin metabolome, biosynthesis and critical role of stereochemistry of specialized pro-resolving mediators (SPMs) in inflammation-resolution: Preparing SPMs for long COVID-19, human clinical trials, and targeted precision nutrition. Semin. Immunol..

[19] Schneider C. (2011). Traumeel—An emerging option to nonsteroidal anti-inflammatory drugs in the management of acute musculoskeletal injuries. Int. J. Gen. Med..

[20] St. Laurent G., Toma I., Seilheimer B., Cesnulevicius K., Schultz M., Tackett M., Zhou J., Ri M., Shtokalo D., Antonets D. (2021). RNAseq analysis of treatment-dependent signaling changes during inflammation in a mouse cutaneous wound healing model. BMC Genom..

[21] Hoch M., Smita S., Cesnulevicius K., Schultz M., Lescheid D., Wolkenhauer O., Gupta S. (2023). Network analyses reveal new insights into the effect of multicomponent Tr14 compared to single-component diclofenac in an acute inflammation model. J. Inflamm..

[22] Jordan P.M., van Goethem E., Müller A.M., Hemmer K., Gavioli V., Baillif V., Burmeister Y., Krömmelbein N., Dubourdeau M., Seilheimer B. (2021). The Natural Combination Medicine Traumeel (Tr14) Improves Resolution of Inflammation by Promoting the Biosynthesis of Specialized Pro-Resolving Mediators. Pharmaceuticals.

[23] González de Vega C., Speed C., Wolfarth B., González J. (2013). Traumeel vs. diclofenac for reducing pain and improving ankle mobility after acute ankle sprain: A multicentre, randomised, blinded, controlled and non-inferiority trial. Int. J. Clin. Pract..

[24] Wolfarth B., Speed C., Raymuev K., Vanden Bossche L., Migliore A. (2022). Managing pain and inflammation associated with musculoskeletal disease: Time for a change?. Curr. Med. Res. Opin..

[25] Duncan J.J., Farr J.E. (1988). Comparison of diclofenac sodium and aspirin in the treatment of acute sports injuries. Am. J. Sports Med..

[26] Morán M. (1991). Double-blind Comparison of Diclofenac Potassium, Ibuprofen and Placebo in the Treatment of Ankle Sprains. J. Int. Med. Res..

[27] Banning M. (2008). Topical diclofenac: Clinical effectiveness and current uses in osteoarthritis of the knee and soft tissue injuries. Expert Opin. Pharmacother..

[28] Derry S., Moore R.A., Gaskell H., McIntyre M., Wiffen P.J. (2015). Topical NSAIDs for acute musculoskeletal pain in adults. Cochrane Database Syst. Rev..

[29] Derry S., Wiffen P.J., Kalso E.A., Bell R.F., Aldington D., Phillips T., Gaskell H., Moore R.A. (2017). Topical analgesics for acute and chronic pain in adults—An overview of Cochrane Reviews. Cochrane Database Syst. Rev..

[30] Wiffen P.J., Xia J. (2020). Systematic review of topical diclofenac for the treatment of acute and chronic musculoskeletal pain. Curr. Med. Res. Opin..

[31] Zell J., Connert W.D., Mau J., Feuerstake G. (1988). Treatment of acute sprains of the ankle joint. Double-blind study assessing the effectiveness of a homeopathic ointment preparation. Fortschr. Med..

[32] Böhmer D., Ambrus P. (1992). Treatment of sports injuries with Traumeel ointment: A controlled double-blind study. Biol. Ther..

[33] van Haselen R. (2017). An integrative review of the evidence for the effectiveness of the antihomotoxic drug Traumeel. RMJ.

[34] (2013). World Medical Association Declaration of Helsinki: Ethical principles for medical research involving human subjects. Jama.

[35] International Council for Harmonisation of Technical Requirements for Pharmaceuticals for Human Use (Ich) (2016). Guidelines for Good Clinical Practice E6 (R2).

[36] Schulz K.F., Altman D.G., Moher D. (2010). *CONSORT* 2010 Statement: Updated guidelines for reporting parallel group randomised trials. BMJ (Clin. Res. Ed.).

[37] Pires R., Pereira A., Abreu E.S.G., Labronici P., Figueiredo L., Godoy-Santos A., Kfuri M. (2014). Ottawa ankle rules and subjective surgeon perception to evaluate radiograph necessity following foot and ankle sprain. Ann. Med. Health Sci. Res..

[38] Bijur P.E., Silver W., Gallagher E.J. (2001). Reliability of the visual analog scale for measurement of acute pain. Acad. Emerg. Med..

[39] Johnson J.R. Standard methods for analysis and reporting of VAS or NRS derived pain relief response scores. Proceedings of the PhUSE 2016.

[40] Martin R.L., Irrgang J.J., Burdett R.G., Conti S.F., Van Swearingen J.M. (2005). Evidence of validity for the Foot and Ankle Ability Measure (FAAM). Foot Ankle Int..

[41] Maurer W., Hothorn L.A., Lehmacher W., Vollmer J. (1995). Multiple comparisons in drug clinical trials and preclinical assays: A-priori ordered hypotheses. Testing Principles in Clinical and Preclinical Trials.

[42] ICH E9 Statistical Principles for Clinical Trials—Scientific Guidelines. https://www.ema.europa.eu/en/ich-e9-statistical-principles-clinical-trials-scientific-guideline.

[43] European Medical Agency Guidelines on the Clinical Development of Medicinal Products Intended for the Treatment of Pain 2016. (EMA/CHMP/970057/2011). https://www.ema.europa.eu/en/documents/scientific-guideline/guideline-clinical-development-medicinal-products-intended-treatment-pain-first-version_en.pdf.

[44] Lacerda D., Pacheco D., Rocha A.T., Diniz P., Pedro I., Pinto F.G. (2023). Current Concept Review: State of Acute Lateral Ankle Injury Classification Systems. J. Foot Ankle Surg..

[45] Olsen M.F., Bjerre E., Hansen M.D., Hilden J., Landler N.E., Tendal B., Hróbjartsson A. (2017). Pain relief that matters to patients: Systematic review of empirical studies assessing the minimum clinically important difference in acute pain. BMC Med..

[46] Gerdesmeyer L., Klueter T., Rahlfs V.W., Muderis M.A., Saxena A., Gollwitzer H., Harrasser N., Stukenberg M., Prehn-Kristensen A. (2017). Randomized Placebo-Controlled Placebo Trial to Determine the Placebo Effect Size. Pain. Physician.

[47] Forsberg J.T., Martinussen M., Flaten M.A. (2017). The Placebo Analgesic Effect in Healthy Individuals and Patients: A Meta-Analysis. Psychosom. Med..

[48] Pabst H., Gruber G., Picciotto R., Barbaro B., Giordan N. (2023). Efficacy and safety of Diclofenac sodium plaster in patients with acute pain of the limbs: A randomized, placebo and active-controlled, double-blind, parallel-group trial. Eur. Rev. Med. Pharmacol. Sci..

[49] Dworkin R.H., Turk D.C., Wyrwich K.W., Beaton D., Cleeland C.S., Farrar J.T., Haythornthwaite J.A., Jensen M.P., Kerns R.D., Ader D.N. (2008). Interpreting the clinical importance of treatment outcomes in chronic pain clinical trials: IMMPACT recommendations. J. Pain.

[50] Srikandarajah S., Gilron I. (2011). Systematic review of movement-evoked pain versus pain at rest in postsurgical clinical trials and meta-analyses: A fundamental distinction requiring standardized measurement. PAIN.

[51] Walls R.J., Ross K.A., Fraser E.J., Hodgkins C.W., Smyth N.A., Egan C.J., Calder J., Kennedy J.G. (2016). Football injuries of the ankle: A review of injury mechanisms, diagnosis and management. World J. Orthop..

[52] Bullock G.S., Murray E., Vaughan J., Kluzek S. (2021). Temporal trends in incidence of time-loss injuries in four male professional North American sports over 13 seasons. Sci. Rep..

[53] Lam K.C., Marshall A.N., Bay R.C., Wikstrom E.A. (2023). Patient-Reported Outcomes at Return to Sport After Lateral Ankle Sprain Injuries: A Report From the Athletic Training Practice-Based Research Network. J. Athl. Train..

[54] Picot B., Lopes R., Rauline G., Fourchet F., Hardy A. (2023). Development and Validation of the Ankle-GO Score for Discriminating and Predicting Return-to-Sport Outcomes after Lateral Ankle Sprain. Sports Health.

[55] Smith M.D., Vicenzino B., Bahr R., Bandholm T., Cooke R., Mendonça L.D.M., Fourchet F., Glasgow P., Gribble P.A., Herrington L. (2021). Return to sport decisions after an acute lateral ankle sprain injury: Introducing the PAASS framework—An international multidisciplinary consensus. Br. J. Sports Med..

[56] Attenborough A.S., Hiller C.E., Smith R.M., Stuelcken M., Greene A., Sinclair P.J. (2014). Chronic ankle instability in sporting populations. Sports Med..

[57] Cooke M.W., Marsh J.L., Clark M., Nakash R., Jarvis R.M., Hutton J.L., Szczepura A., Wilson S., Lamb S.E., group C.t. (2009). Treatment of severe ankle sprain: A pragmatic randomised controlled trial comparing the clinical effectiveness and cost-effectiveness of three types of mechanical ankle support with tubular bandage. The CAST trial. Health Technol. Assess..

[58] De Boer A.S., Schepers T., Panneman M.J., Van Beeck E.F., Van Lieshout E.M. (2014). Health care consumption and costs due to foot and ankle injuries in the Netherlands, 1986-2010. BMC Musculoskelet. Disord..

[59] Knowles S.B., Marshall S.W., Miller T., Spicer R., Bowling J.M., Loomis D., Millikan R.W., Yang J., Mueller F.O. (2007). Cost of injuries from a prospective cohort study of North Carolina high school athletes. Inj. Prev..

[60] Gan T.J. (2010). Diclofenac: An update on its mechanism of action and safety profile. Curr. Med. Res. Opin..

[61] Chen Y., Lyu K., Lu J., Jiang L., Zhu B., Liu X., Li Y., Liu X., Long L., Wang X. (2022). Biological response of extracorporeal shock wave therapy to tendinopathy in vivo (review). Front. Vet. Sci..

