# Supplementary material for: Topical Treatment Is Effective and Safe for Acute Ankle Sprains: The Multi-Center Double-Blind Randomized Placebo-Controlled TRAUMED Trial
Source: J Clin Med. 2024 Feb 1;13(3):841. doi: 10.3390/jcm13030841 (PMC10856131; doi:10.3390/jcm13030841)

TRAUMED trial – Supplementary tables and figures

Supplementary Table S1: Composition of the investigational medicinal products (IMP)

Tr14 gel	
Ingredient	Traumed ® gel, 100 g contains:
Arnica Montana D3	1.500g
Calendula officinalis Ø	0.450g
Hamamelis virginiana Ø	0.450g
Echinacea Ø	0.150g
Echinacea purpurea Ø	0.150g
Matricaria recutita Ø	0.150g
Symphytum officinale D4	0.100g
Bellis perennis Ø	0.100g
Hypericum perforatum D6	0.090g
Achillea millefolium Ø	0.090g
Aconitum napellus D1	0.050g
Atropa belladonna D1	0.050g
Mercurius solubilis Hahnemanni D6	0.040g
Hepar sulfuris D6	0.025g
Excipients	Purified water, ethanol, carbomers, sodium hydroxide solution
Ethanol content	24.4% (V/V)
Placebo gel	
Ingredient	Placebo gel, 100g contains:
Excipients	Purified water, ethanol, carbomers, sodium hydroxide solution
Ethanol content	24.4% (V/V)
Diclofenac gel	
Ingredient	Diclofenac Heumann gel, (1%), 100g contains:

Diclofenac Sodium Salt	1.00g
Excipients	Purified water, ethanol, isopropanol, carbomers, ammonia solution

Supplementary Table S2: Trial milestones

Date	Milestone	Comment
7 June 2017	Registration in EU Clinical Trial Registry	First entered in the EudraCT database
22 August 2017	Registration in EU Clinical Trial Registry	Start date in EudraCT = date, the trial was authorized to proceed
5 January 2018	Protocol approval by Regulatory Authorities	BfArM approves Protocol version 3.0
22 January 2018	Protocol approval by Central Ethics Committee	CEC approves Protocol version 3.0
26 February 2018	Recruitment starts	FPFV
18 December 2019 – 17 February 2020	First recruitment interruption	Due to IMP expiry and resupply delay
3 March 2020	Protocol Amendment	BfArM approves Protocol Amendment (version 4.0) to the Protocol version 3.0 with sample size enhancement to 808 = Protocol version 4.0
17 March 2020	Protocol approval by Central Ethics Committee	CEC approves protocol version 4.0
23 March 2020 – 25 June 2020	Second recruitment interruption	Due to SARS-CoV-2 pandemic
18 November 2020	Recruitment ends	LPLV
9 March 2021	Blind Data Review Meeting	
25 March 2021	Database hard lock	Global end of the trial date in EudraCT
20 April 2021	Statistical Analysis Plan finalized	The decision to introduce Day 7 as an additional primary endpoint in the chain of *a priori* hypotheses. The original endpoints on Day 4 remained unchanged.
30 April 2021	Data unblinding	The data unblinding request was signed. The generation of unblinded analysis sets started on May 3rd, 2021, and was completed by May 10, 2021.
31 January 2022	Final Clinical Trial Report approved	Including appendices
4 October 2022	Results posted in EU Clinical Trial Registry	Status in EudraCT: posted

BfArM: Bundesinstitut für Arzneimittel und Medizinprodukte (German Federal Institute for Drugs and Medical Devices); CEC: Central Ethics Committee;
FPFV: First patient first visit; LPLV: Last patient last visit

Supplementary Table S3: Median values of AUC, absolute scores, and percent-change-from-baseline for VAS scores for pain on passive movement (VASPM), LOCF

**Median (full range) area under the curve (AUC) for pain on passive movement in Visual Analog Scale (VAS mm)**					
Endpoint values	FAS			PP	
Number of subjects analyzed	Tr1 gel N= 316	Placebo gel N =155		Tr14 gel (N = 314)	Diclofenac gel (N = 146)
Day 2 (Visit 2)	69.00 (30.40-114.40)	70.51 (39.97-97.51)		68.88 (30.40-114.40)	70.58 (34.71-98.85)
Day 4 (Visit 3)	187.88 (51.46-370.73)	200.75 (86.08-374.43)		187.50 (51.46-370.73)	197.19 (76.74-366.54)
Day 7 (Visit 4)	294.14 (63.46-592.47)	353.42 (101.28- 620.07)		293.85 (63.46-592.47)	327.93 (94.76- 637.79)
Day 14 (Visit 5)	394.65 (66.96-1126.39)	577.00 (101.28-1050.77)		394.37 (66.96-1126.39)	505.39 (98.76-1266.54)
**Median (full range) absolute values for pain on passive movement in Visual Analog Scale (VAS mm)**					
Day 1 (Visit 1)	74.0 (51.0-100)	74.0 (51.0-100)		74.0 (51.0-100)	75.0 (53.0-100)
Day 2 (Visit 2)	65.0 (2.0-100)	69.0 (18.0-100)		65.0 (2.0-100)	67.0 (12.0-95.0)
Day 4 (Visit 3)	45.0 (2.0-86.0)	54.0 (2.0-92.0)		45.0 (2.0-86.0)	50.5 (1.0-88.0)
Day 7 (Visit 4)	22.0 (0.0-81.0)	40.0 (0.0-83.0)		22.0 (0.0-81.0)	33.5 (0.0-92.0)
Day 14 (Visit 5)	5.0 (0.0-76.0)	18.0 (0.0-80.0)		5.0 (0.0-76.0)	10.0 (0.0-88.0)
**Median (full range) percentage-change-from-baseline for pain on passive movement in Visual Analog Scale**					
Day 2 (Visit 2)	-11.11 ((-96.67)-27.45)	-6.49 ((-75.00)-20.51)		-11.11 ((-96.67)-27.45)	-8.28 ((-87.63)-15.28)
Day 4 (Visit 3)	-39.32 ((-97.92)-13.43)	-25.93 ((-97.40)-25.00)		-39.61 ((-97.92)-13.33)	-28.17 ((-98.82)-11.67)
Day 7 (Visit 4)	-68.275 ((-100.00)-13.43)	-45.68 ((-100.00)-17.65)		-68.88 ((-100.00)-(-3.95))	-55.66 ((-100.00)-3.70)
Day 14 (Visit 5)	-93.17 ((-100.00)-13.43)	-77.27 ((-100.0)-(-1.96))		-93.33 ((-100.00)-5.560)	-84.98 ((-100.00)-9.43)

Supplementary Table S4: Parametric ANCOVA analysis VAS AUC scores for pain on passive movement (AUCPM) for comparisons Tr14 vs placebo (FAS) and Tr14 vs diclofenac (PP).

AUCPM Visit 2/Day 2 General Model		
	Tr14 vs placebo (FAS)	Tr14 vs diclofenac (PP)
Mean 1	69.3393	69.2242
Mean 2	70.0596	68.8593
MeanDiff	0.7203	-0.3649
Valid N1	316	314
Valid N2	155	146
LB	-0.6635	-1.937105099
UB	2.10414036	1.207305099
P	0.2962	0.6359
AUCPM Visit 3/Day4 General Model		
Mean 1	193.5814	192.9532
Mean 2	203.8071	196.0949
MeanDiff	10.2257	3.1417
Valid N1	316	314
Valid N2	155	146
LB	1.3873	-5.959303339
UB	19.06411424	12.24270334
P	0.0205	0.4812
AUCPM Visit 4/Day 7 General Model		
Mean 1	305.4616	304.0339
Mean 2	348.9655	329.8989
MeanDiff	43.5039	25.865
Valid N1	316	314
Valid N2	155	146
LB	25.777955461.229844555	7.934457942
UB	61.22984455	43.79554206
P	< 0.0001	0.0034
AUCPM Visit 5/Day 14 General Model		
Mean 1	438.0048	433.6797
Mean 2	557.2234	505.4429
MeanDiff	119.2186	71.7632
Valid N1	316	314
Valid N2	155	146
LB	81.60812247	34.2222148
UB	156.8290775	109.3041852
P	< 0.0001	0.0001

AUCPM: Area under the curve for passive movement; FAS: Full Analysis Set; PP: Per Protocol analysis set

Supplementary Table S5: Median values of AUC, absolute scores, and percent-change-from-baseline for VAS scores on pain at rest (VASRS). LOCF

**Median (full range) Area Under the Curve (AUC) for pain at rest in Visual Analog Scale (VAS mm)**					
Endpoint values	FAS			PP	
Number of subjects analyzed	Tr14gel (N = 316)	Placebo gel (N = 155)		Tr14d gel (N = 314)	Diclofenac gel (N = 146)
Day 2 (Visit 2)	28.025 (0-107.4375)	31.43 (0-91.5)		27.41 (0.0-107.436)	24.87 (2.6-86.50)
Day 4 (Visit 3)	65.9 (2.48-305.50)	73.4 (2.49-259.22)		65.4 (2.48-305.50)	58.49 (6.67-272.50)
Day 7 (Visit 4)	90.31 (2.48-570.5)	109.83 (2.49-504.30)		90.11 (2.49-570.5)	89.99 (11.52-435.50)
Day 14 (Visit 5)	116.06 (2.48-895.45)	159.74 (2.49-896.49)		114.57 (2.48-895.45)	128.89 (12.5-872.0)
**Median (full range) absolute values for pain at rest in Visual Analog Scale (VAS mm)**					
Day 1 (Visit 1)	32.0 (0.0-95.0)	35.0 (0.0-100.0)		31.0 (0.0-95.0)	26.5 (4.0-96.0)
Day 2 (Visit 2)	21.5 (0.0-97.0)	23.0 (0.0-84.0)		21.0 (0.0-97.0)	20.5 (1.0-85.0)
Day 4 (Visit 3)	12.0 (0.0-87.0)	16.0 (0.0-85.0)		12.0 (0.0-87.0)	12.0 (0.0-74.0)
Day 7 (Visit 4)	4.5 (0.0-75.0)	9.0 (0.0-67.0)		4.0 (0.0-75.0)	7.0 (0.0-63.0)
Day 14 (Visit 5)	0.0 (0.0-60.0)	2.0 (0.0-73.0)		0.0 (0.0-58.0)	1.0 (0.0-62.0)
**Median (full range) percent-change-from-baseline for pain at rest in Visual Analog Scale**					
Day 2 (Visit 2)	-15.29 ((-100.00)-100.00)	-9.09 ((-98.44)-37.50)		-15.38 ((-100.00)-100.00)	-12.05 ((-98.86))-170.00)
Day 4 (Visit 3)	-50.00 ((-100.00)-162.500)	-36.86 ((-100.00)-100.00)		-50.00 ((-100.00)-162.50)	-42.86 ((-100.00)-66.67)
Day 7 (Visit 4)	-82.47 ((-100.00)-10.940)	-63.49 ((-100.00)-280.00)		-82.63 ((-100.00)-10.94)	-73.51 ((-100.00)-20.00)
Day 14 (Visit 5)	-100.00 ((-100.00)-9.430)	-92.16 ((-100.00)-50.00)		-100.00 ((-100.00)-0.00)	-97.43 ((-100.00)-40.00)

Supplementary Table S6: Median values of absolute scores, and percent-change-from-baseline for FAAM-ADL scores. LOCF

Median (full range) absolute values for FAAM-ADL					
Endpoint values	FAS			PP	
Number of subjects analyzed	Tr14 gel (N = 316)	Placebo gel (N = 155)		Tr14 gel (N = 314)	Diclofenac gel (N = 146)
Day 1 (Visit 1)	52.38 (0.0-95.24)	50.0 (0.0-97.62)		52.38 (0.0-95.24)	52.08 (0.0-97.62)
Day 2 (Visit 2)	47.42 (1.19-92.86)	46.43 (14.29-94.05)		47.22 (1.19-92.86)	46.43 (7.14-94.05)
Day 4 (Visit 3)	30.95 (1.19-89.29)	35.71 (0.0-91.67)		30.95 (1.19-89.29)	32.14 (0.0-90.48)
Day 7 (Visit 4)	17.11 (0.0-88.16)	26.19 (0.0-66.67)		16.89 (0.0-88.16)	21.24 (0.0-94.05)
Day 14 (Visit 5)	4.76 (0.0-76.19)	9.52 (0.0-61.90)		4.76 (0.0-76.19)	7.14 (0.0-70.24)
**Median (full range) percent-change-from-baseline for FAAM-ADL**					
Day 2 (Visit 2)	-7.69 ((-51.72)-233.33)	-1.33 ((-65.96))-625.00)		-7.69 ((-51.72)-233.33)	-4.495 ((-67.65)-30.00)
Day 4 (Visit 3)	-37.74 ((-87.50)-77.78)	-22.86 ((-100.00)-325.00)		-38.10 ((-87.50)-77.78)	-32.99 ((-100.00)-16.00)
Day 7 (Visit 4)	-63.77 ((-100.00)-35.63)	-46.34 ((-100.00)-225.00)		-64.29 ((-100.00)-35.63)	-59.57 ((-100.00)-1.28)
Day 14 (Visit 5)	-90.00 ((-100.00)-25.49)	-78.57 ((-100.00)-17.65)		-90.18 ((-100.00)-25.49)	-86.67 ((-100.00)-1.72)

FAAM-ADL: Foot and Ankle Ability Measure - Activities of Daily Living

Supplementary Figure S1: Disposable Dosing Card

The color of the Tr14 gel differed slightly from that of diclofenac and placebo gels (diclofenac gel was indistinguishable from the placebo gel). The design of the dosing card camouflaged the color of the IMP to secure the blinding. It was approved by the German Federal Institute for Drugs and Medical Devices (BfArM) as sufficient to ensure a double-blind design of the trial.

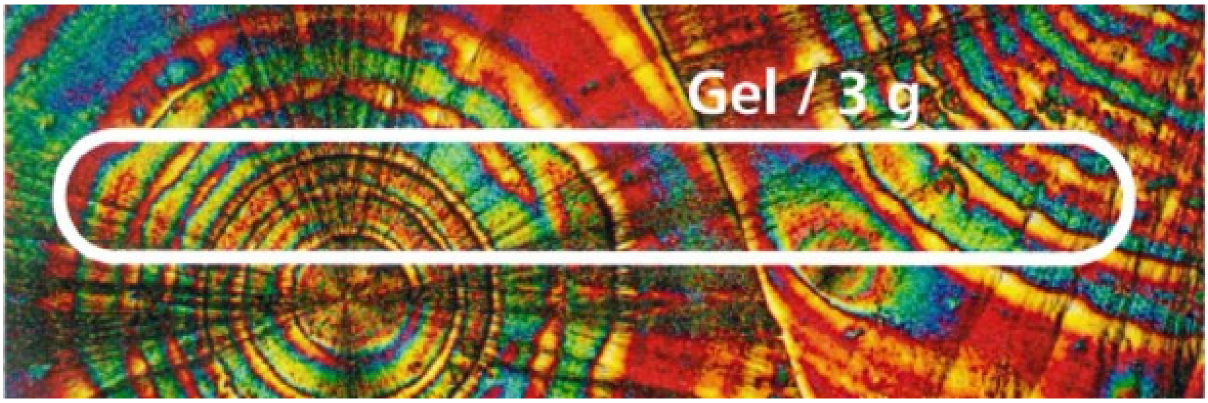

Supplementary Figure S2: Pain on passive movement (VASPM) at all visits

A: Boxplot showing AUC for pain on passive movement in VAS for all visits (median, percentile 10-90, LOCF) for Tr14 vs placebo (FAS).

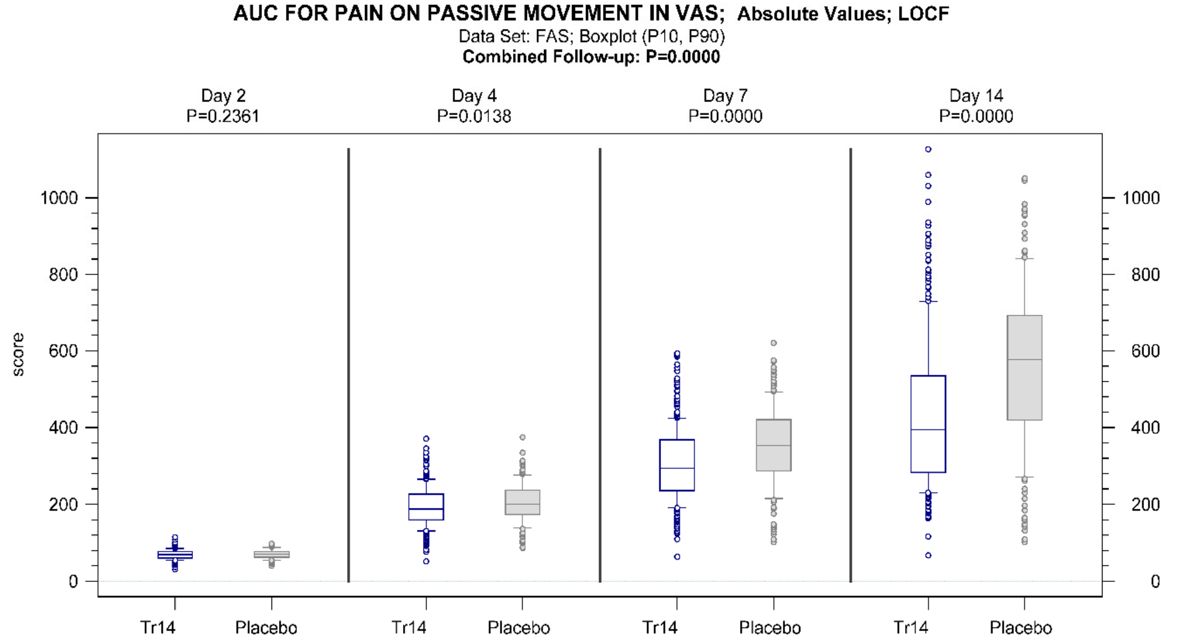

B: Boxplot showing AUC for pain passive movement in VAS for all visits (median, percentile 10-90, LOCF) for Tr14 vs diclofenac (PP).

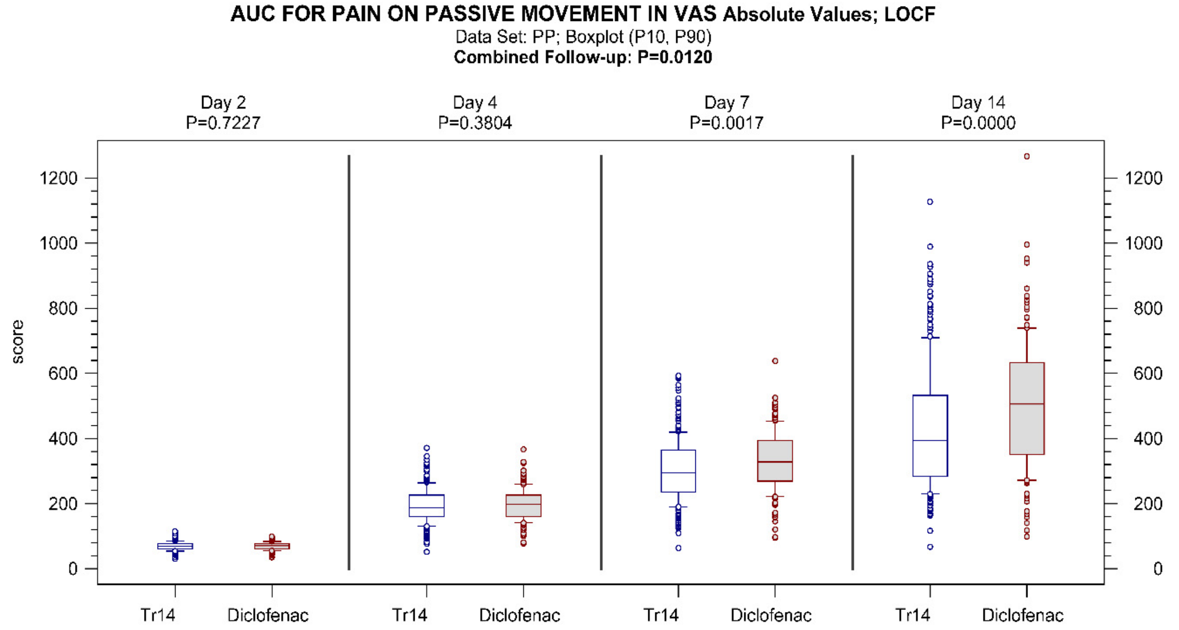

C: Boxplot show percentage change from baseline in pain scores on passive movement for all visits (median, percentile 10-90, LOCF) for Tr14 vs placebo (FAS).

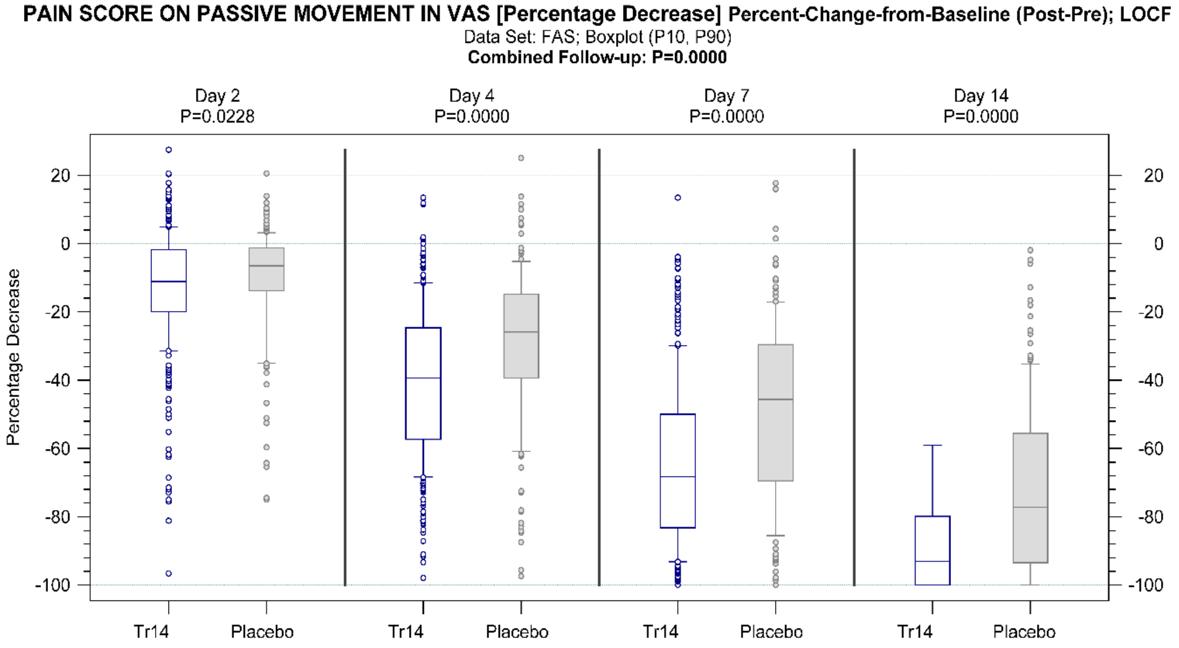

D: Boxplot showing percentage change from baseline in pain scores on passive movement for all visits (median, percentile 10-90, LOCF) for Tr14 vs diclofenac (PP).

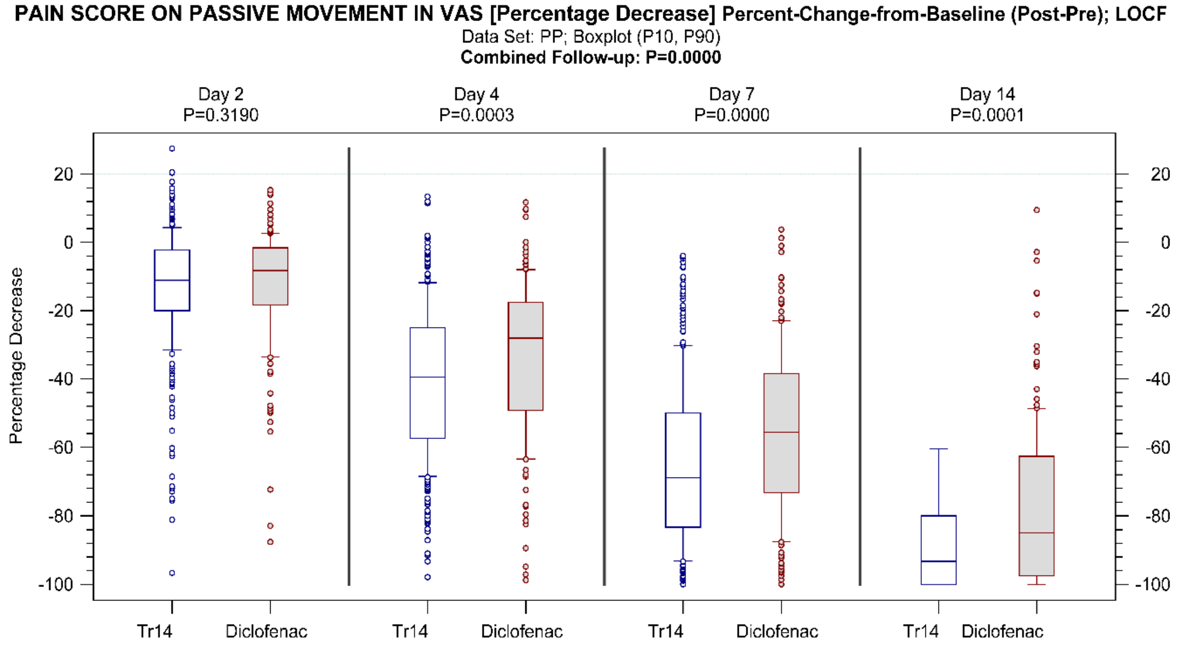

E: Effect size for pain on passive movement in VAS: Absolute values Tr14 vs placebo for all visits (two sided Wilcoxon-Mann-Whitney U-test, FAS, 95% Cl).

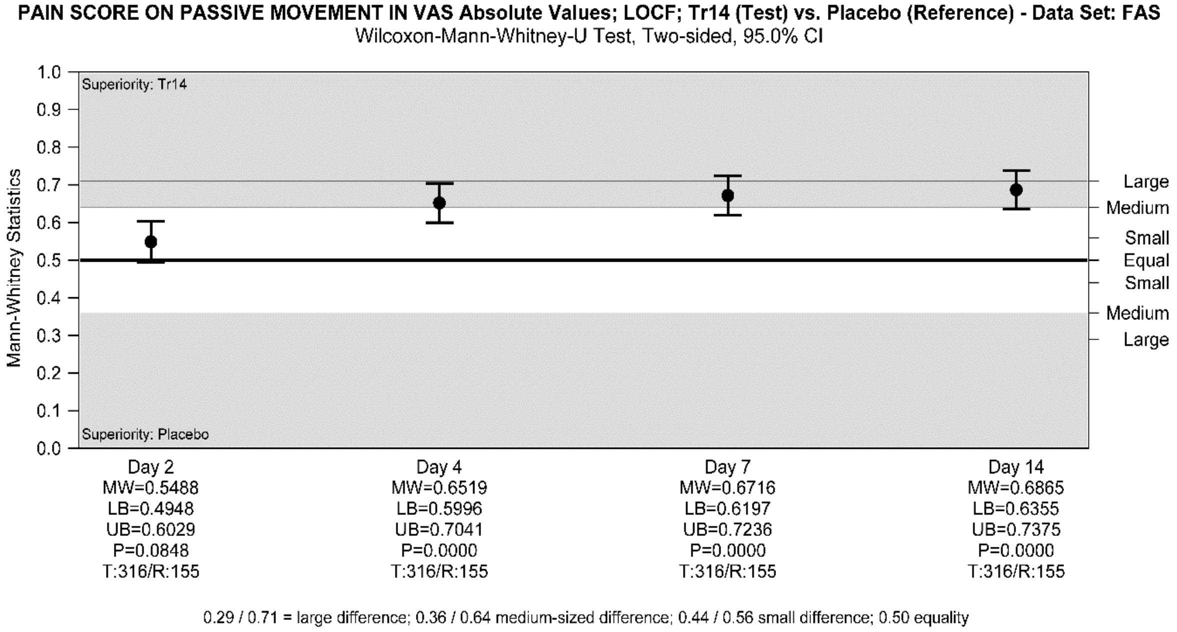

F: Effect size for pain on passive movement in VAS: Absolute values Tr14 vs diclofenac for all visits (two sided Wilcoxon-Mann-Whitney U-test, PP, 95% Cl).

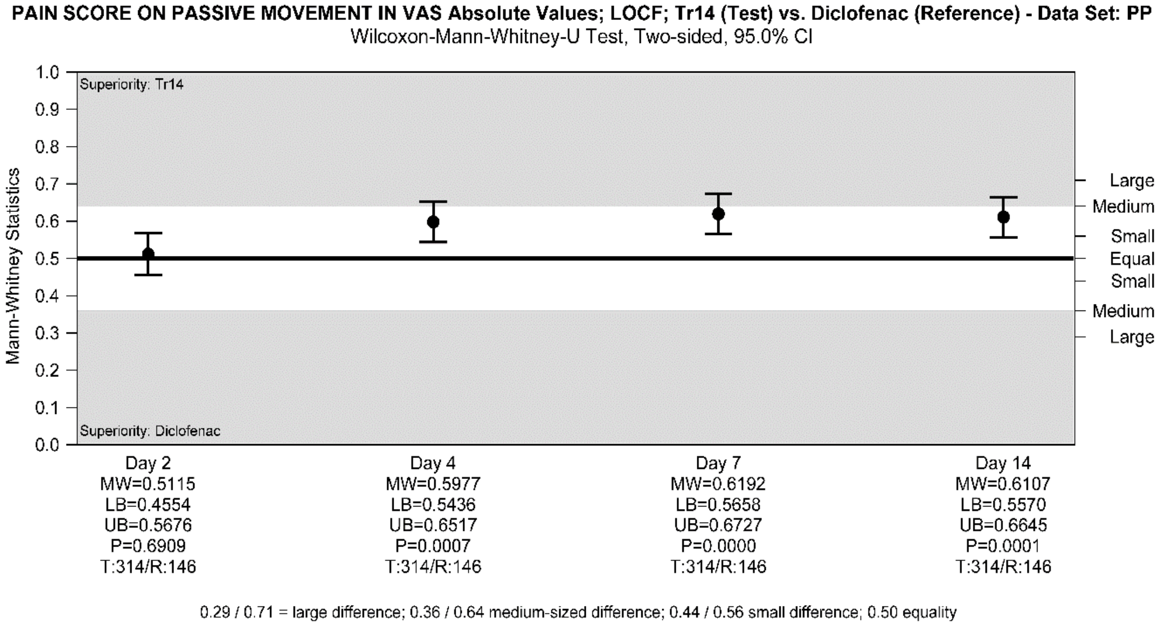

G: Effect size for percentage change from baseline for pain on passive movement in VAS: Tr14 vs placebo for all visits (two sided Wilcoxon-Mann-Whitney U-test, FAS, 95% Cl).

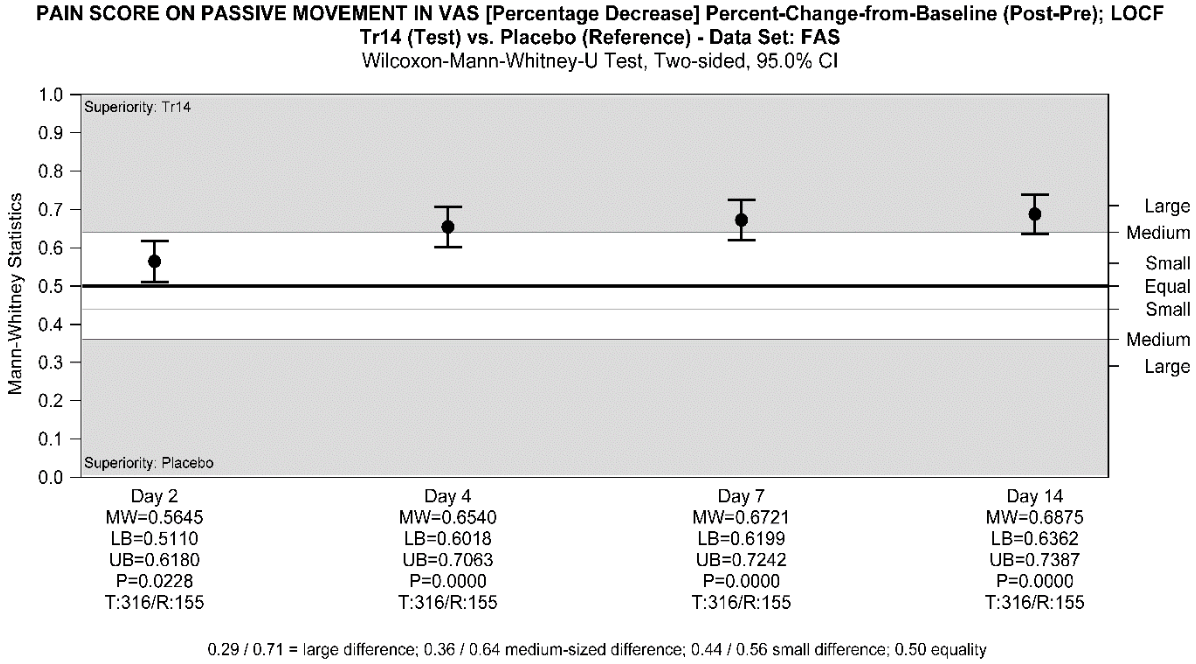

H: Effect size for percentage change from baseline for pain on passive movement in VAS: Tr14 vs diclofenac for all visits (two sided Wilcoxon-Mann-Whitney U-test, PP, 95% Cl).

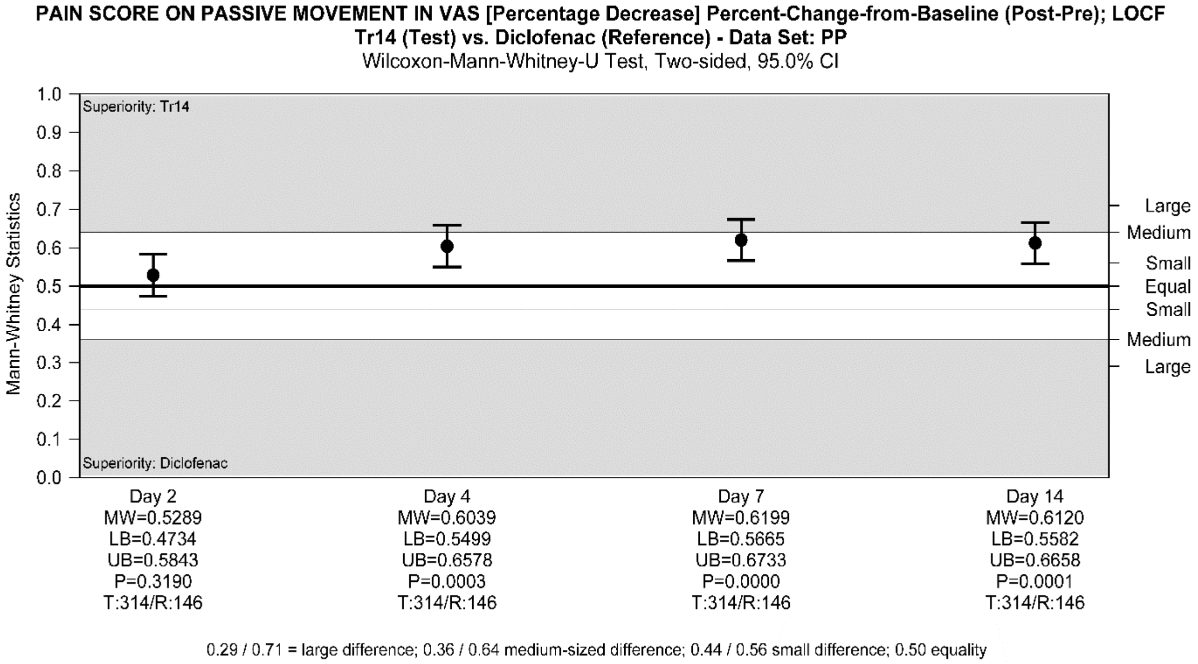

Abbreviations: VAS = Visual analogue scale, LOCF = Last observation carried forward, FAS = Full Analysis Set, CI = Confidence interval, MW = Mann-Whitney estimator, LB = Lower bound of the two-sided confidence interval, UB = Upper bound of the two-sided confidence

interval, P = p-value of Wilcoxon-Mann-Whitney test, T = Valid number of Tr14 group (Test), R = Valid number of placebo group (reference)

Supplementary Figure S3: Pain at rest (VASRS) at all visits

A: Boxplot showing AUC for pain at rest in VAS for all visits (median, percentile 10-90, LOCF) for Tr14 vs placebo (FAS).

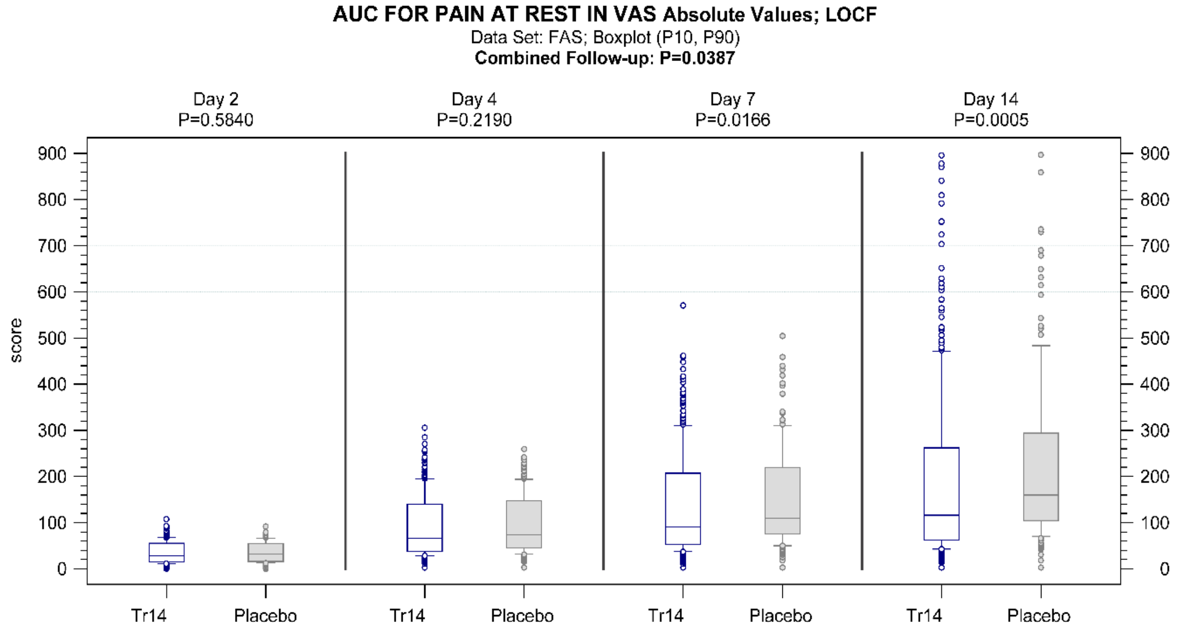

B: Boxplot showing AUC for pain at rest in VAS for all visits (median, percentile 10-90, LOCF) for Tr14 vs diclofenac (PP).

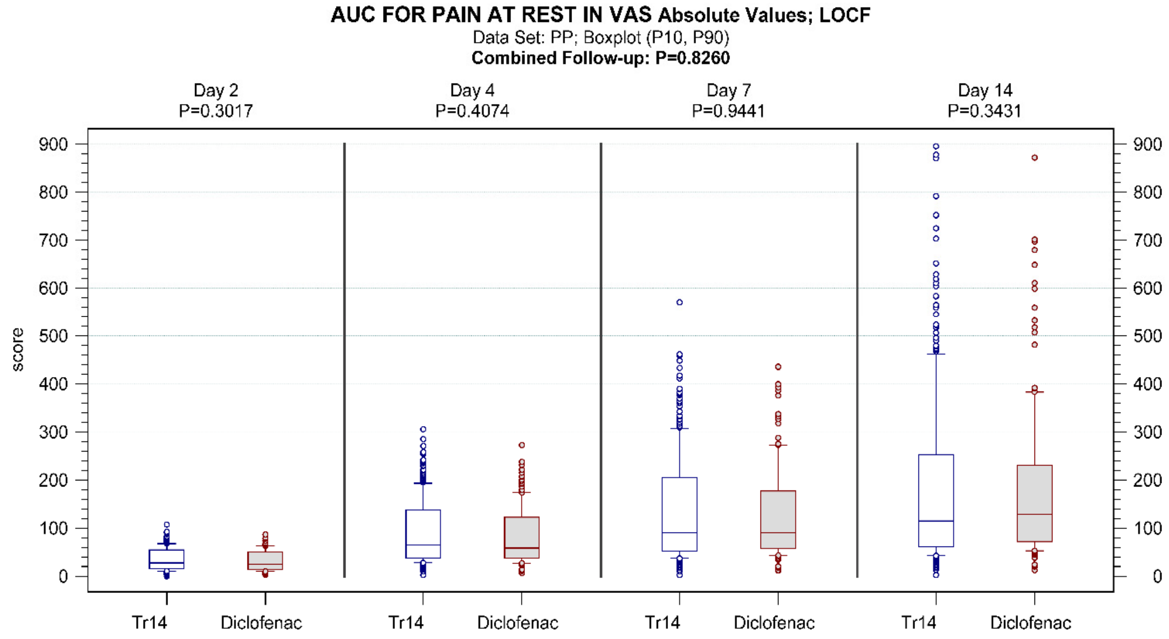

C: Boxplot showing percentage change from baseline in pain scores at rest for all visits (median, percentile 10-90, LOCF) for Tr14 vs placebo (FAS).

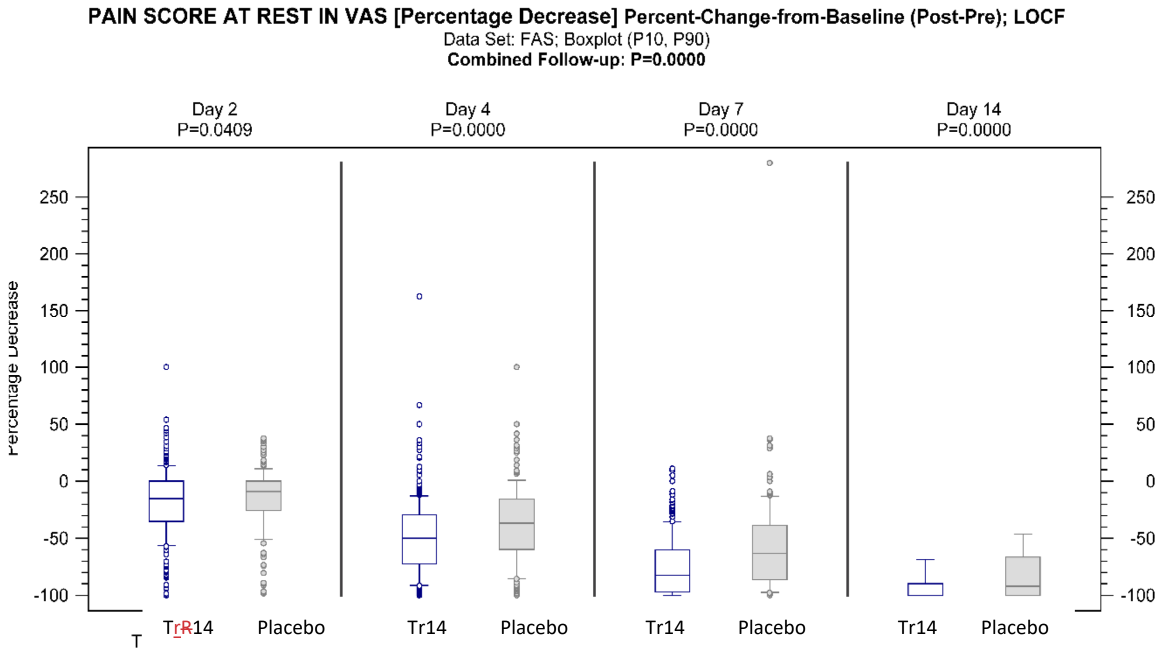

D: Boxplot showing percentage change from baseline in pain scores at rest for all visits (median, percentile 10-90, LOCF) for Tr14 vs diclofenac (PP).

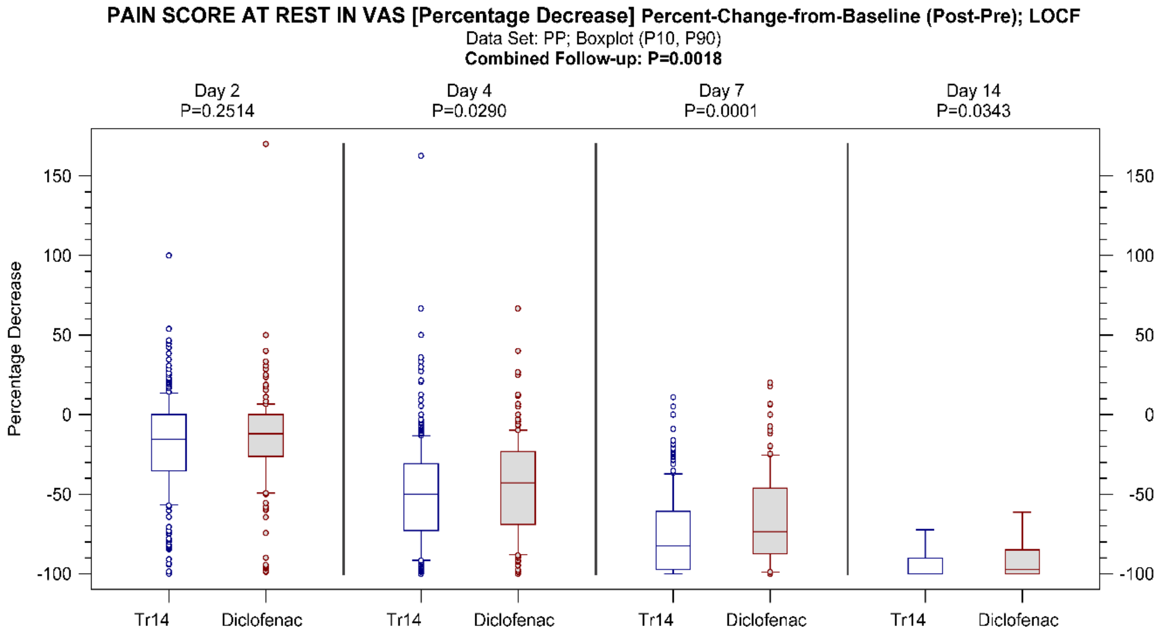

E: Effect size for percentage change from baseline for pain at rest in VAS: Tr14 vs placebo for all visits (two sided Wilcoxon-Mann-Whitney U-test, FAS, 95% Cl).

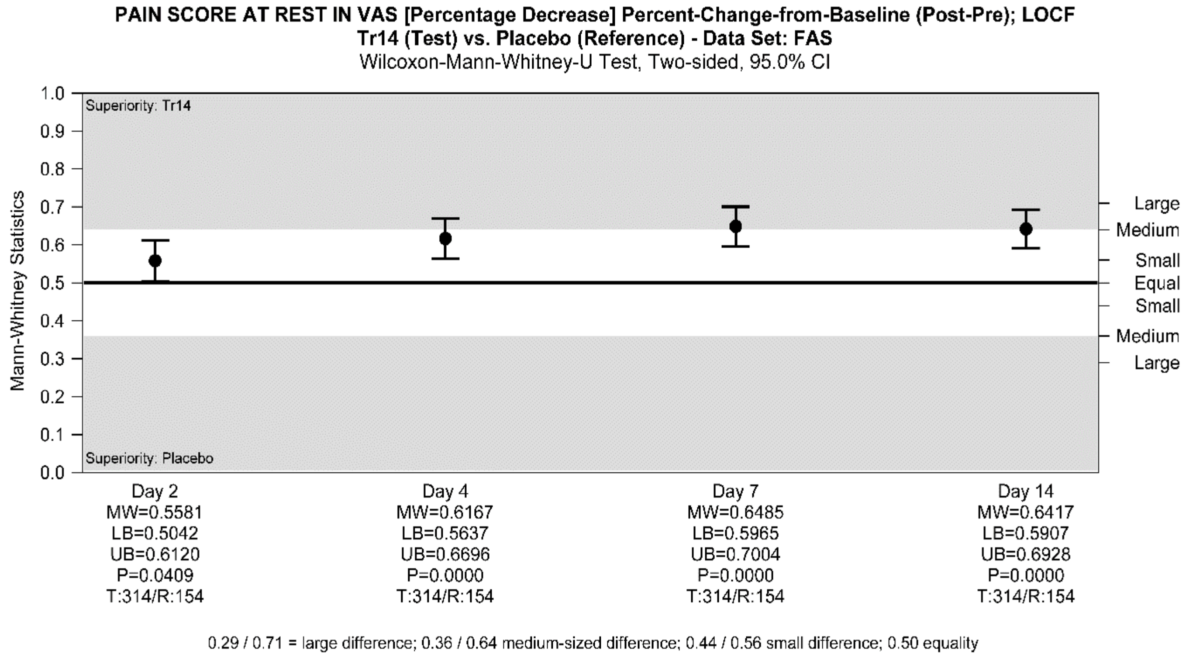

F: Effect size for percentage change from baseline for pain at rest in VAS: Tr14 vs diclofenac for all visits (two sided Wilcoxon-Mann-Whitney U-test, PP, 95% Cl).

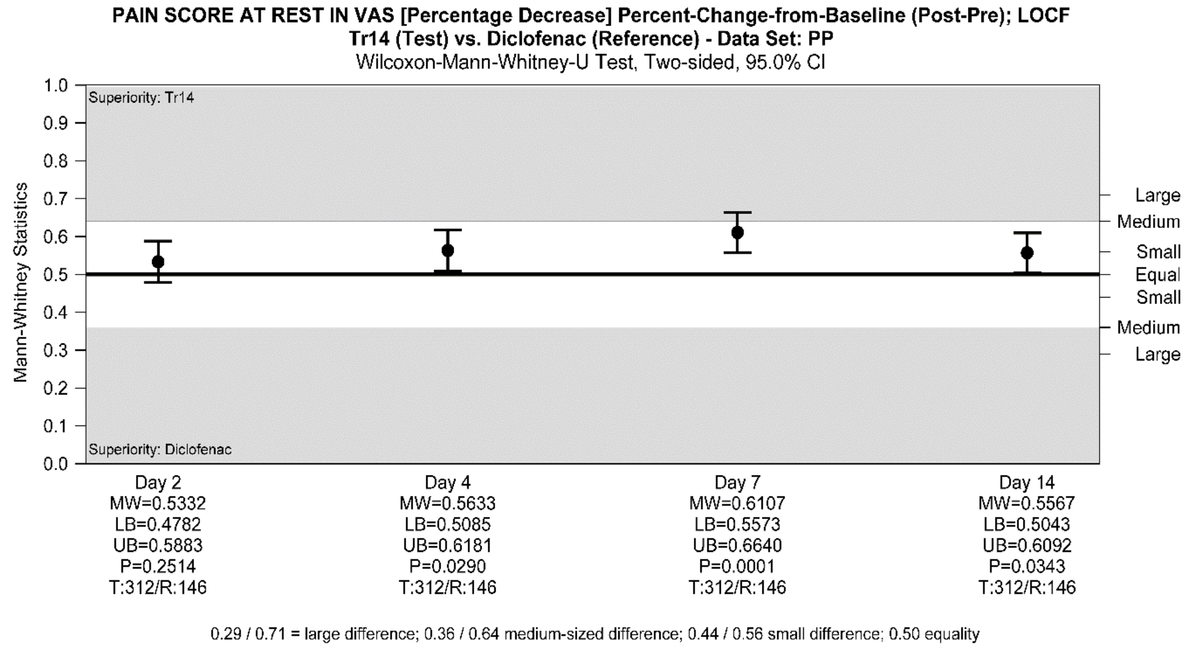

Abbreviations: VAS = Visual analogue scale, LOCF = Last observation carried forward, FAS = Full Analysis Set, CI = Confidence interval, MW = Mann-Whitney estimator, LB = Lower Bound of the two-sided confidence interval, UB = Upper bound of the two-sided confidence interval, P = p-value of Wilcoxon-Mann-Whitney test, T = Valid number of Tr14 group (Test), R = Valid number of placebo group (reference)

Supplementary Figure S4: Foot and Ankle Ability Measure - Activities of Daily Living (FAAM-AD) at all visits

A: Boxplot shows absolute FAAM-ADL subscale values (LOCF, P10/P90) for all visits: Tr14 vs placebo (FAS)

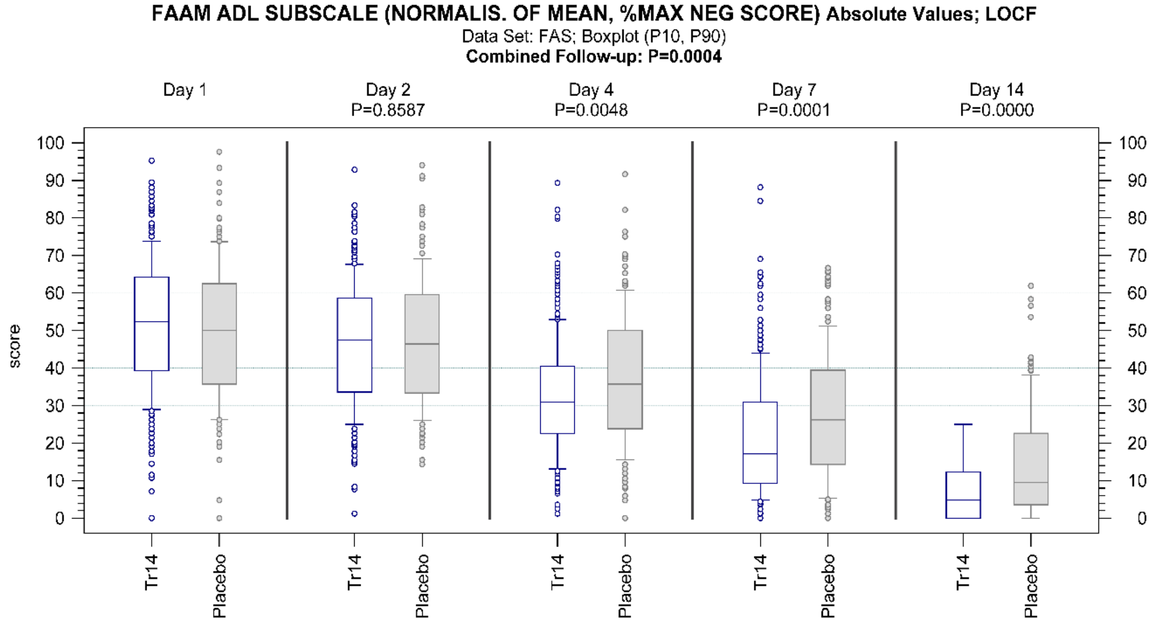

B: Boxplot shows absolute FAAM-ADL subscale values (LOCF, P10/P90) for all visits: Tr14 vs diclofenac (PP).

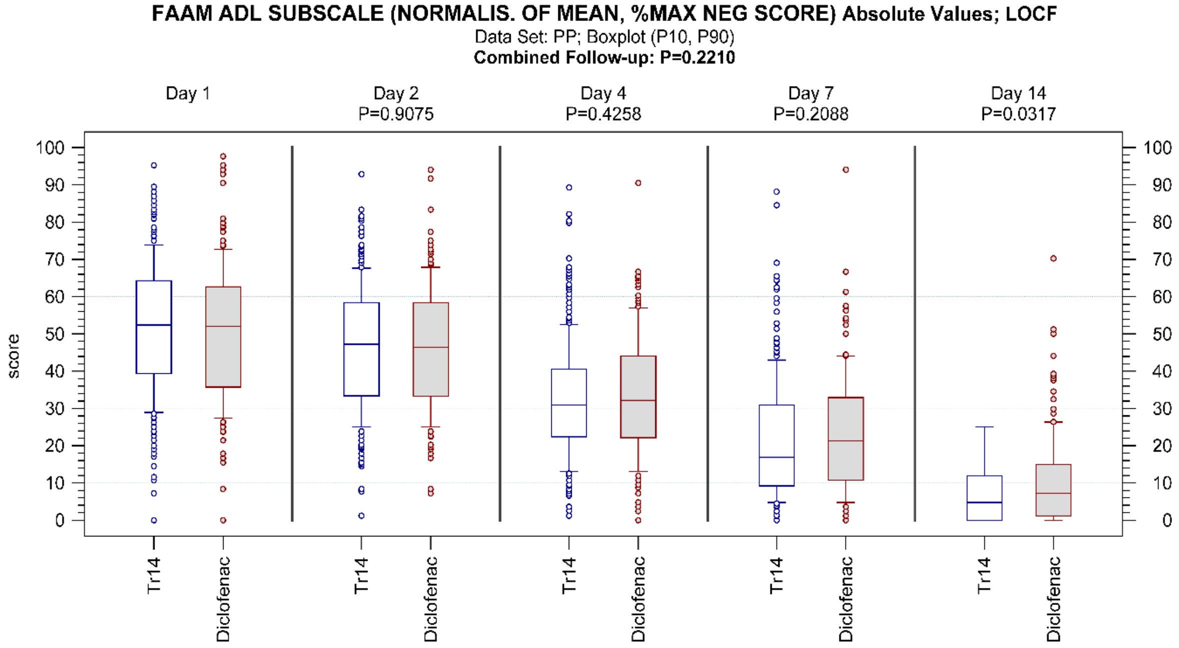

Abbreviations: FAAM-ADL = Foot and Ankle Ability Measure – Activities of Daily Living, LOCF = Last observation carried forward, FAS = Full Analysis Set, PP = Per Protocol set.

Supplementary Figure S5: Kaplan-Meier function for time in days to a 50% reduction in absolute VAS scores for pain on passive movement (VASPM) and pain at rest (VASRS).

A: Time in days to a 50% reduction in absolute VASPM for TR14 vs placebo.

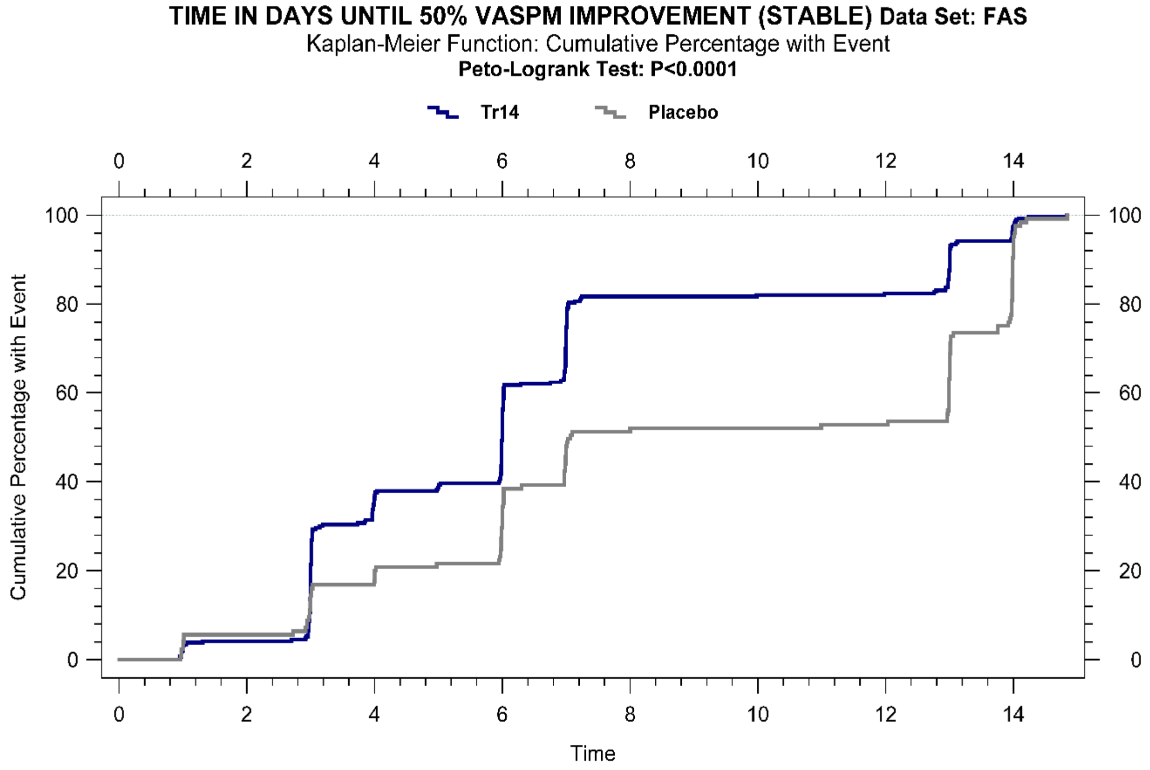

B: Time in days to a 50% reduction in absolute VASPM for TR14 vs diclofenac.

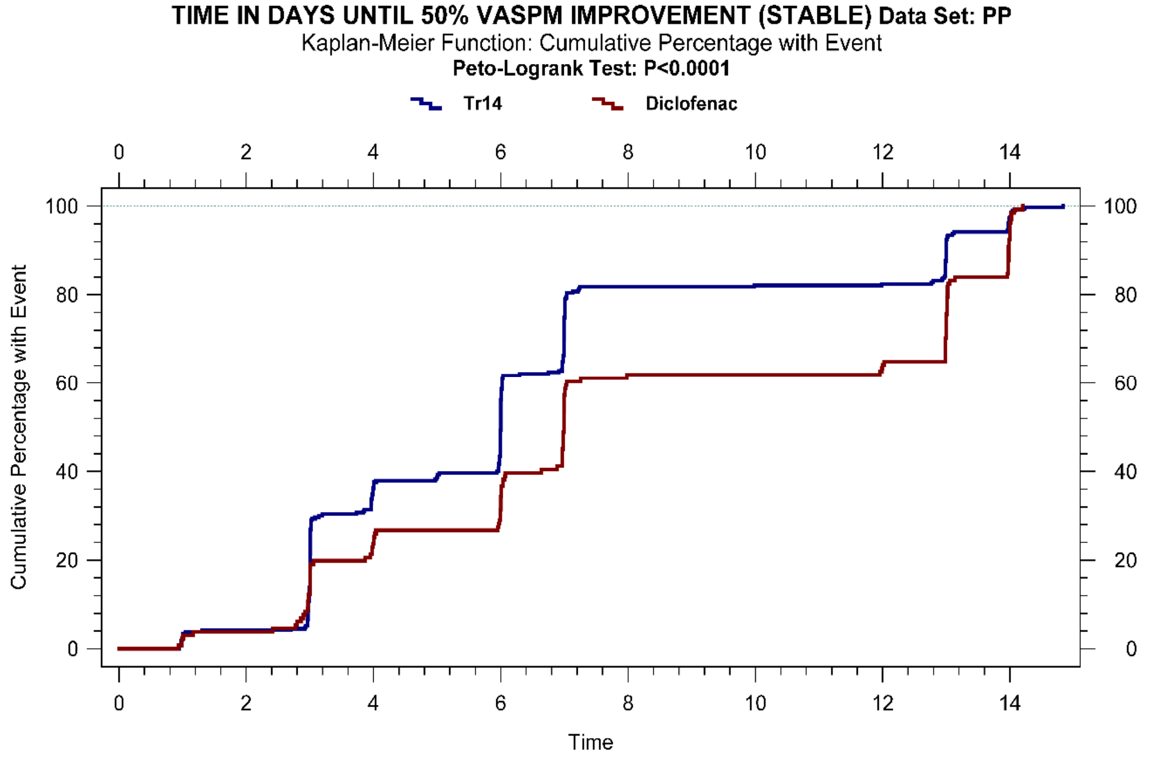

C: Time in days to a 50% reduction in absolute VASRS for TR14 vs placebo.

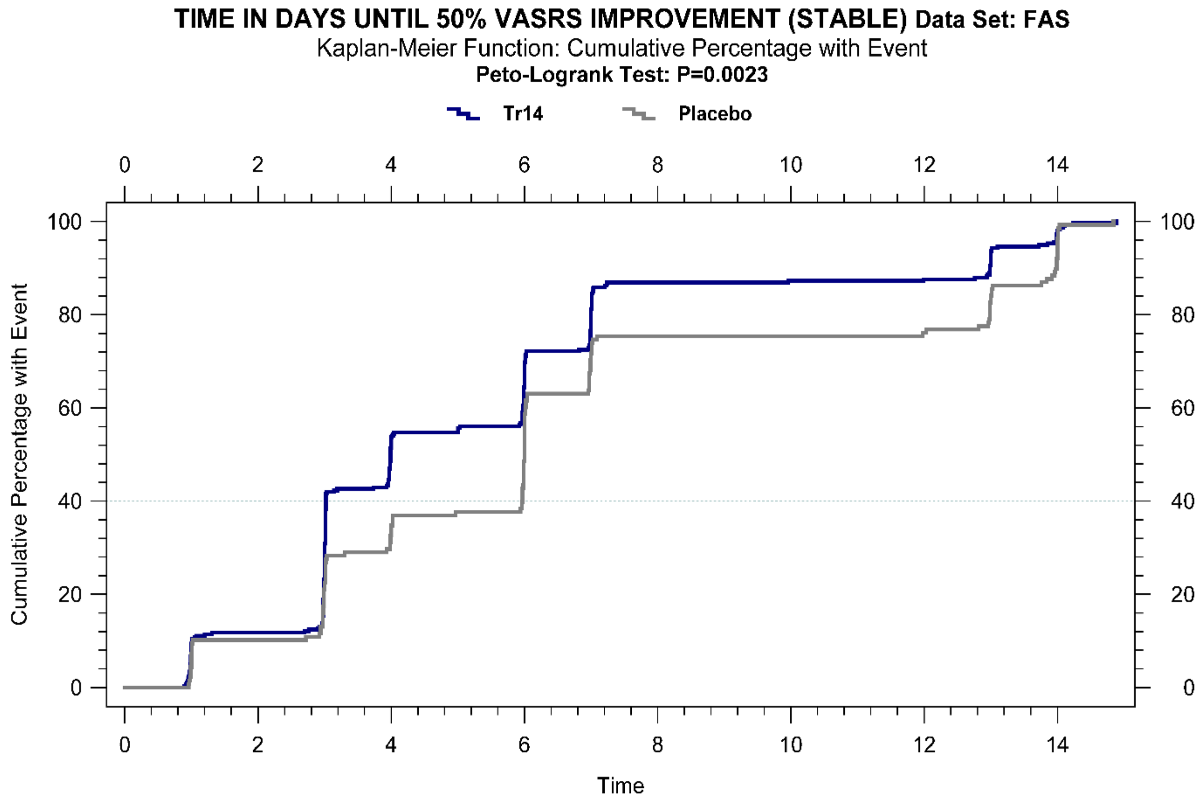

D: Time in days to a 50% reduction in absolute VASRS for TR14 vs diclofenac.

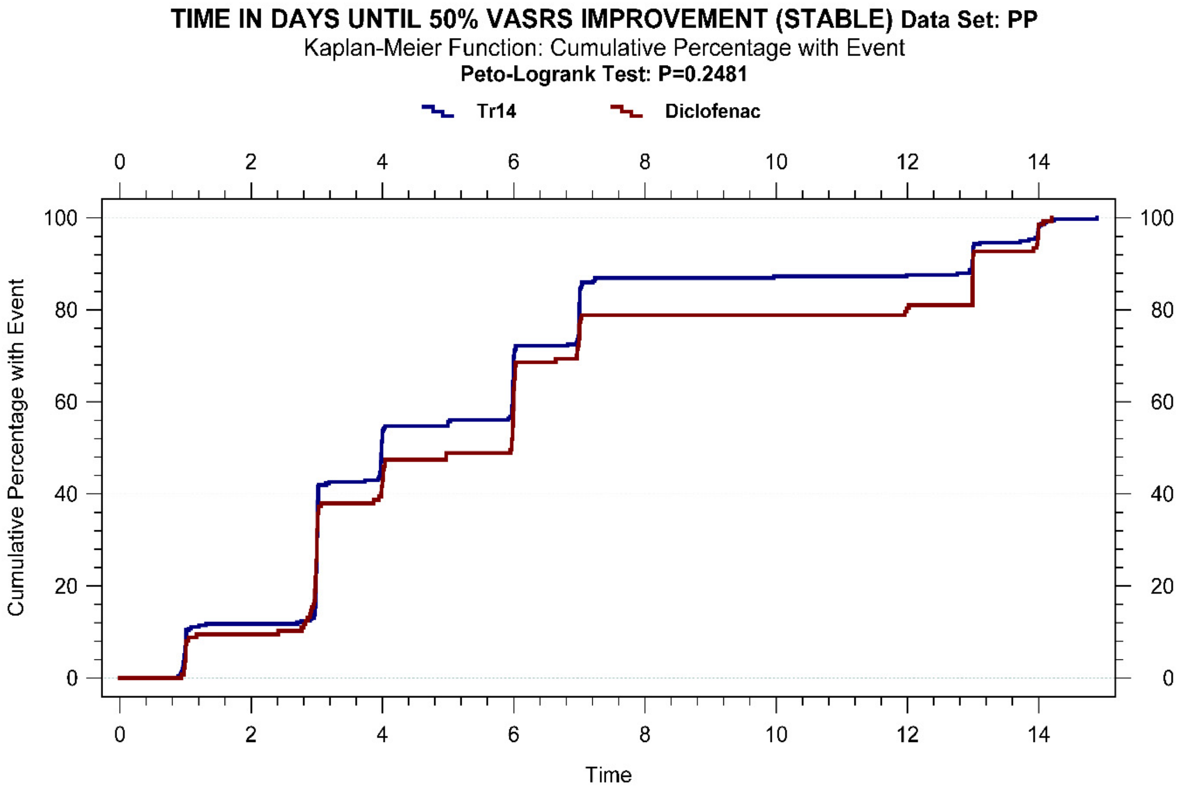

Abbreviations: VASPM = VAS for pain on passive movement; VASRS = VAS for pain at rest; FAS = Full Analysis Set; PP = Per Protocol set.

Supplementary Figure S6: Time in days to 50% reduction of pain at rest in absolute VAS scores.

A: Boxplots show absolute values in days for time to 50% reduction of pain at rest for all visits (Median, Percentile 10-90, LOCF) for Tr14 vs placebo (FAS).

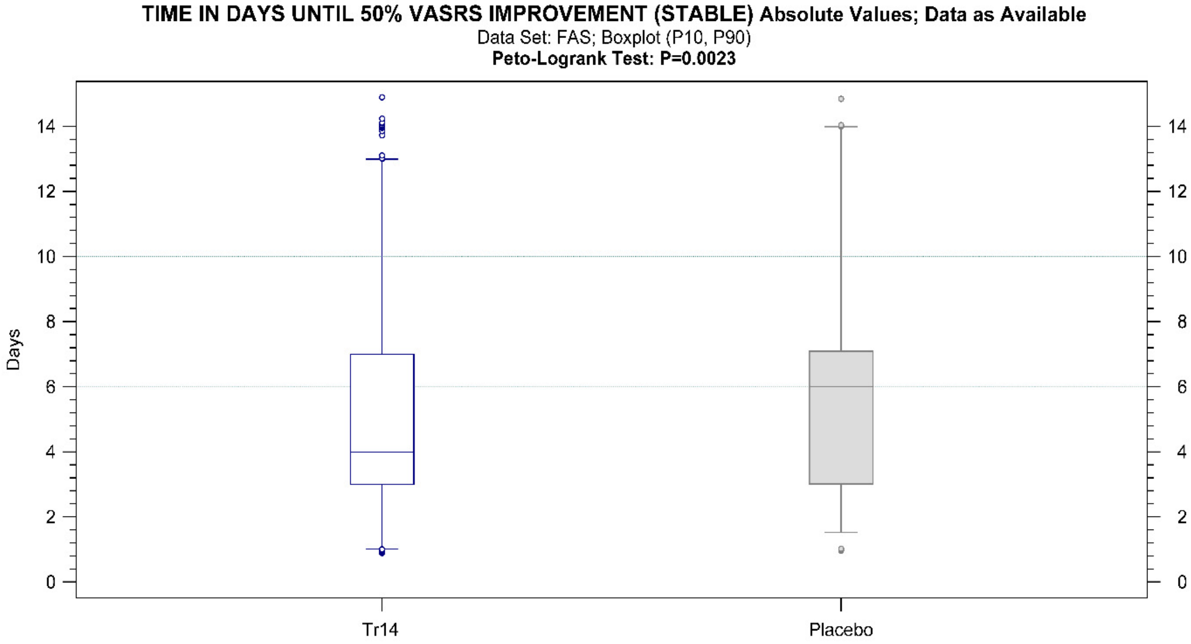

B: Boxplots show absolute values in days for time to 50% reduction of pain at rest for all visits (Median, Percentile 10-90, LOCF) for Tr14 vs diclofenac (PP).

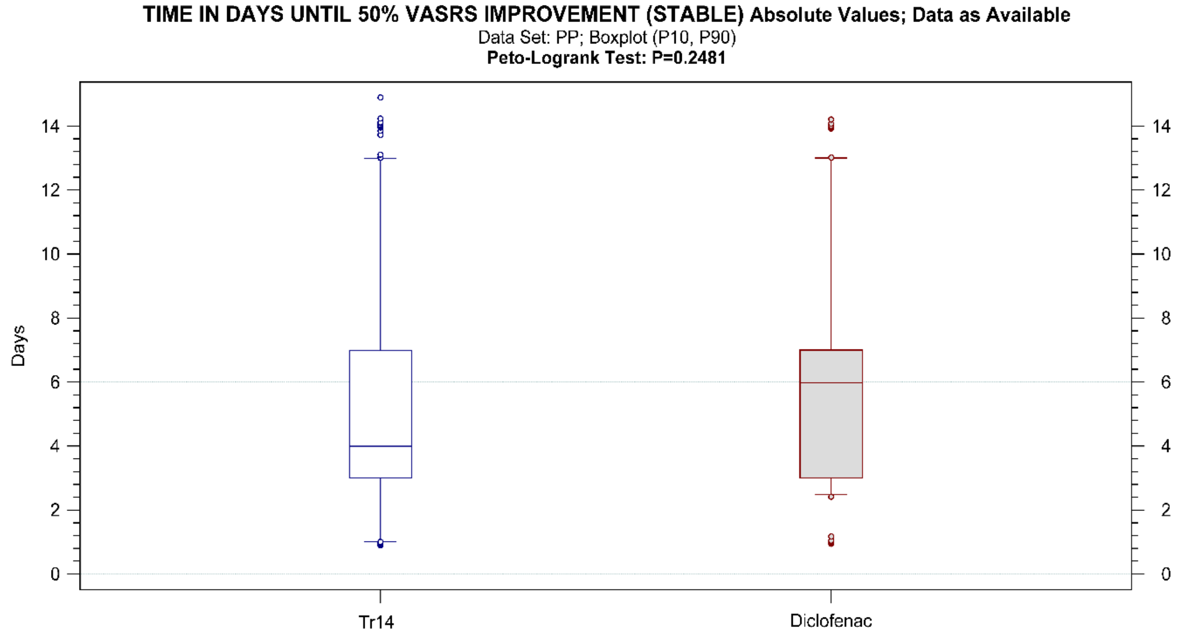

Abbreviations: LOCF = Last observation carried forward, FAS = Full Analysis Set; PP = Per Protocol set.

Supplementary Figure S7: *Post-hoc* responder analysis for 30% and 50% pain reduction for Tr14 gel compared to placebo gel.

VAS pain responder 30% decrease at Day 4

Study or Subgroup	Experimental Events	Experimental Total	Control Events	Control Total	Weight	Risk Difference IV, Fixed, 95% CI
TRAUMED	211	316	63	155	100.0%	0.26 [0.17, 0.35]
**Total (95% CI)**		**316**		**155**	**100.0%**	**0.26 [0.17, 0.35]**
Total events	211		63			
Heterogeneity: Not applicable						
Test for overall effect: Z = 5.50 (P < 0.00001)						

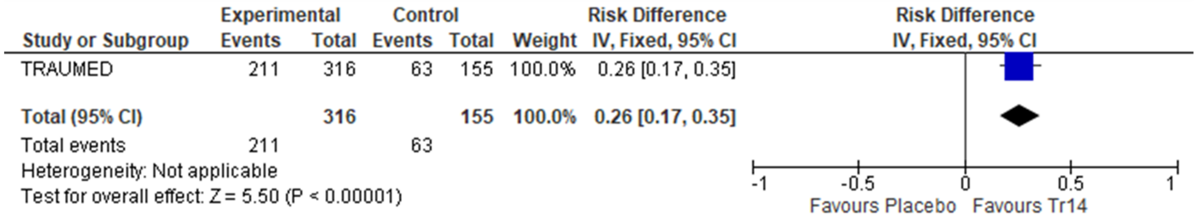

VAS pain responder 30% decrease at Day 7

Study or Subgroup	Experimental Events	Experimental Total	Control Events	Control Total	Weight	Risk Difference IV, Fixed, 95% CI
TRAUMED	284	316	115	155	100.0%	0.16 [0.08, 0.23]
**Total (95% CI)**		**316**		**155**	**100.0%**	**0.16 [0.08, 0.23]**
Total events	284		115			
Heterogeneity: Not applicable						
Test for overall effect: Z = 4.02 (P < 0.0001)						

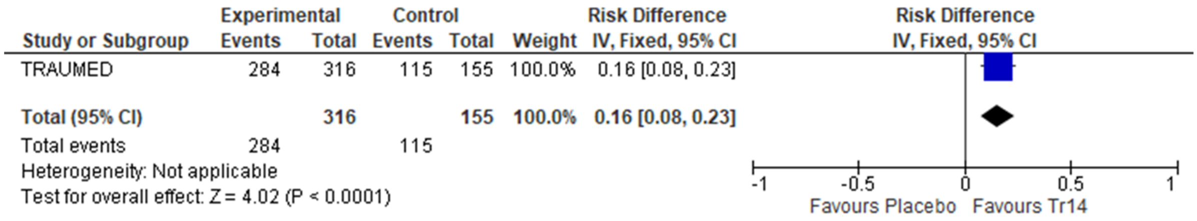

VAS pain responder 50% decrease at Day 4

Study or Subgroup	Experimental Events	Experimental Total	Control Events	Control Total	Weight	Risk Difference IV, Fixed, 95% CI
TRAUMED	112	316	28	155	100.0%	0.17 [0.09, 0.25]
**Total (95% CI)**		**316**		**155**	**100.0%**	**0.17 [0.09, 0.25]**
Total events	112		28			
Heterogeneity: Not applicable						
Test for overall effect: Z = 4.24 (P < 0.0001)						

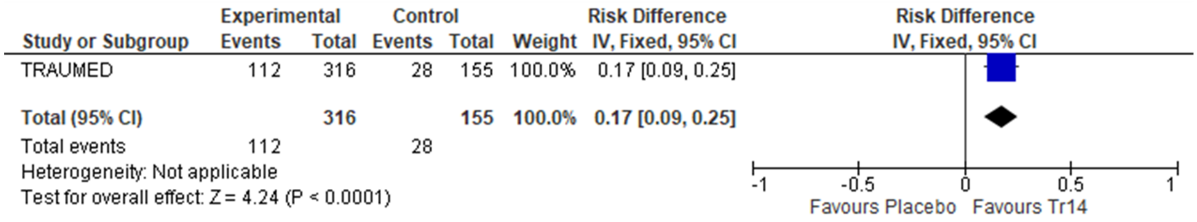

VAS pain responder 50% decrease at Day 7

Study or Subgroup	Experimental Events	Experimental Total	Control Events	Control Total	Weight	Risk Difference IV, Fixed, 95% CI
TRAUMED	238	316	66	155	100.0%	0.33 [0.24, 0.42]
**Total (95% CI)**		**316**		**155**	**100.0%**	**0.33 [0.24, 0.42]**
Total events	238		66			
Heterogeneity: Not applicable						
Test for overall effect: Z = 7.03 (P < 0.00001)						

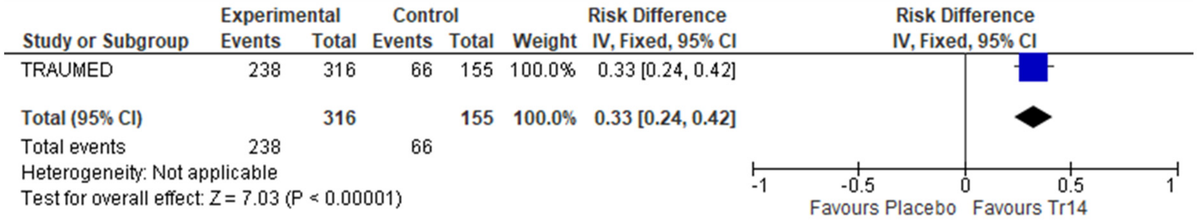

Supplement: Supplementary file 1 [file jcm-13-00841-s001.zip › jcm-2769025-supplementary.pdf]
